# Supplementary figures and images for: The structural repertoire of Fusarium oxysporum f. sp. lycopersici effectors revealed by experimental and computational studies
Source: eLife. 2024 Feb 27;12:RP89280. doi: 10.7554/eLife.89280 (PMC10942635; doi:10.7554/eLife.89280)

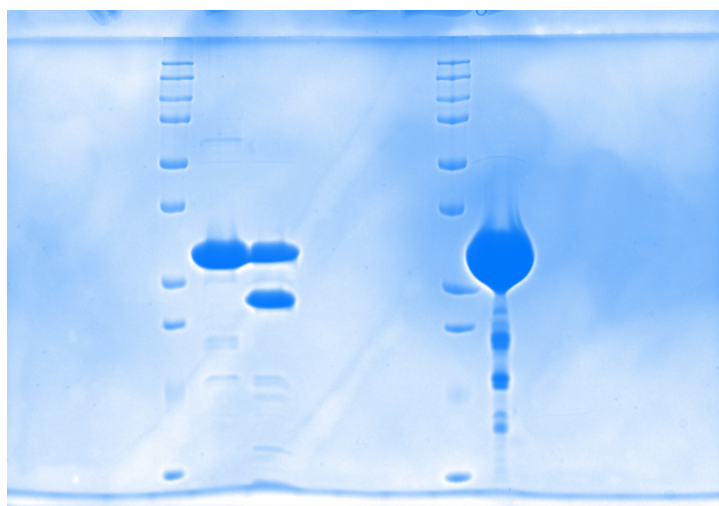

Supplement: Figure 1—figure supplement 1—source data 1. [file elife-89280-fig1-figsupp1-data1.pdf]

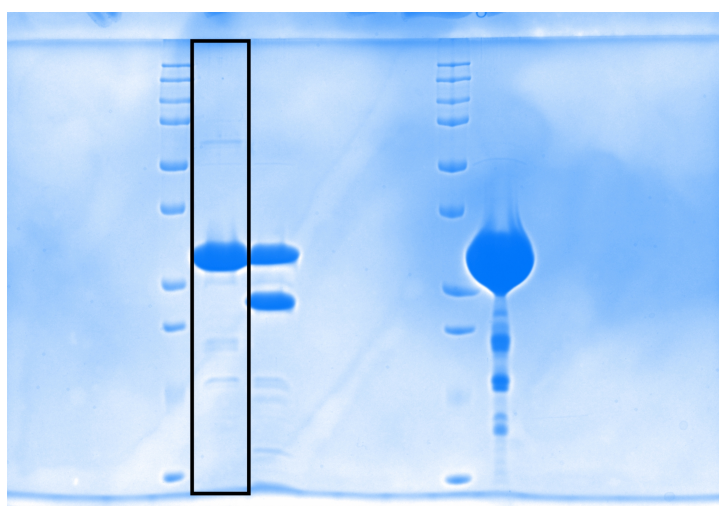

Supplement: Figure 1—figure supplement 1—source data 2. [file elife-89280-fig1-figsupp1-data2.pdf]

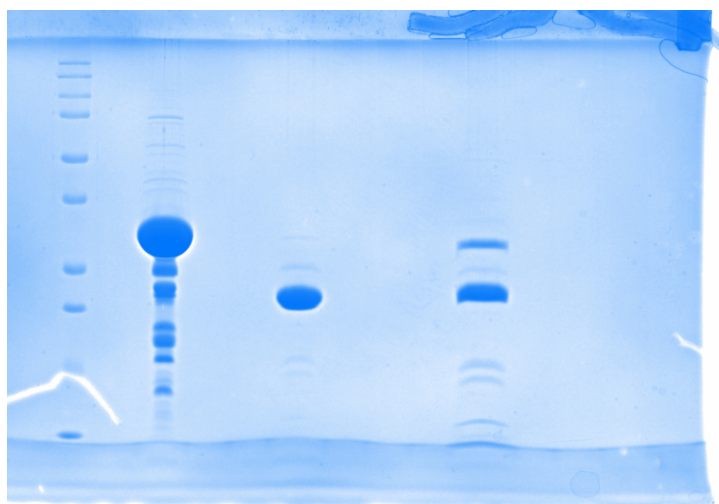

Supplement: Figure 1—figure supplement 1—source data 3. [file elife-89280-fig1-figsupp1-data3.pdf]

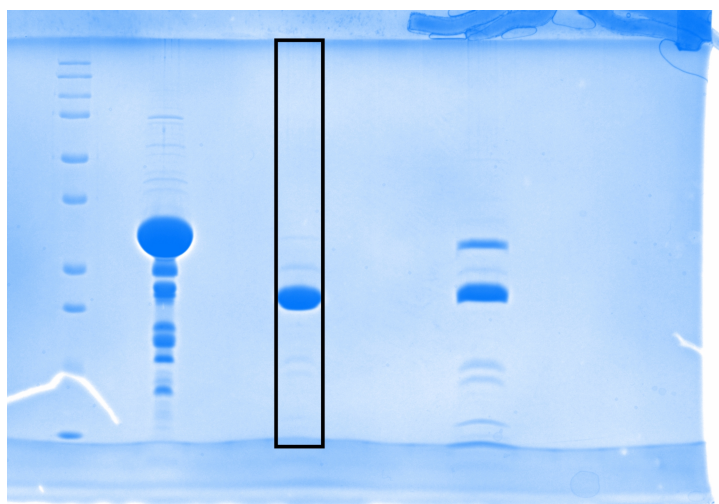

Supplement: Figure 1—figure supplement 1—source data 4. [file elife-89280-fig1-figsupp1-data4.pdf]

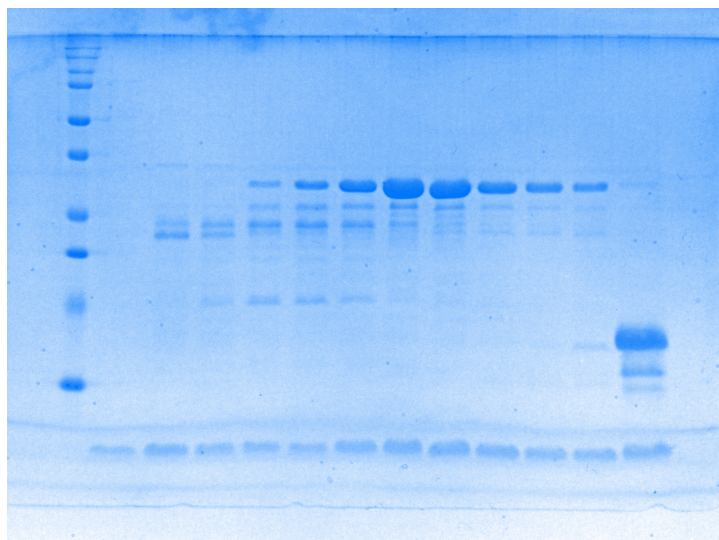

Supplement: Figure 1—figure supplement 1—source data 5. [file elife-89280-fig1-figsupp1-data5.pdf]

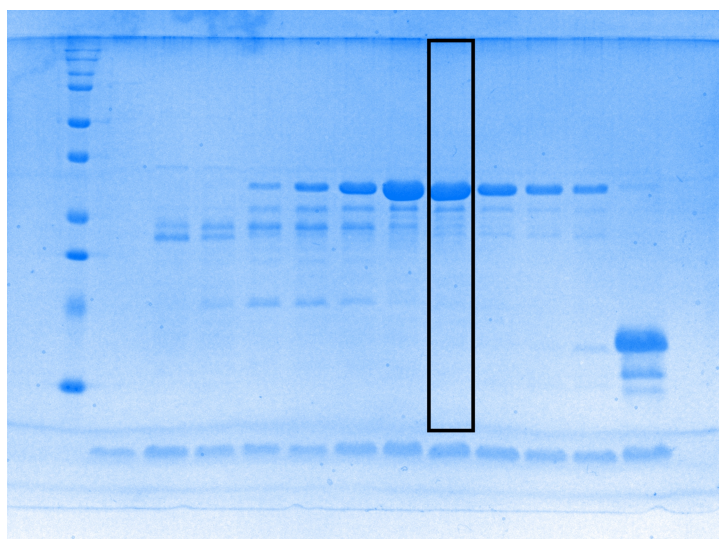

Supplement: Figure 1—figure supplement 1—source data 6. [file elife-89280-fig1-figsupp1-data6.pdf]

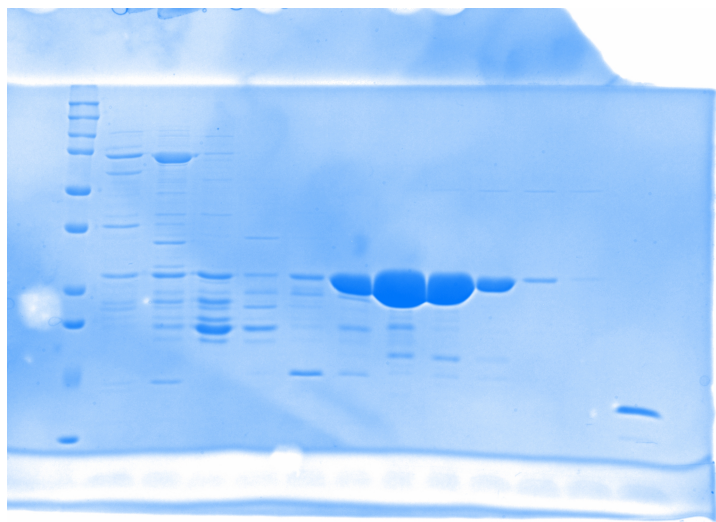

Supplement: Figure 1—figure supplement 1—source data 7. [file elife-89280-fig1-figsupp1-data7.pdf]

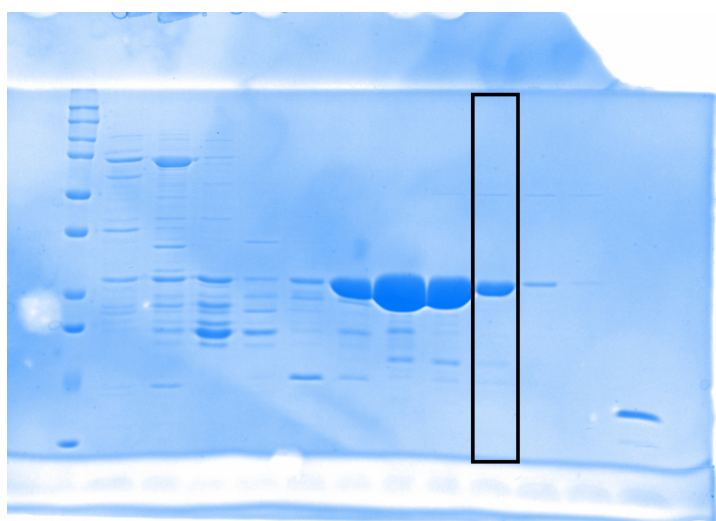

Supplement: Figure 1—figure supplement 1—source data 8. [file elife-89280-fig1-figsupp1-data8.pdf]

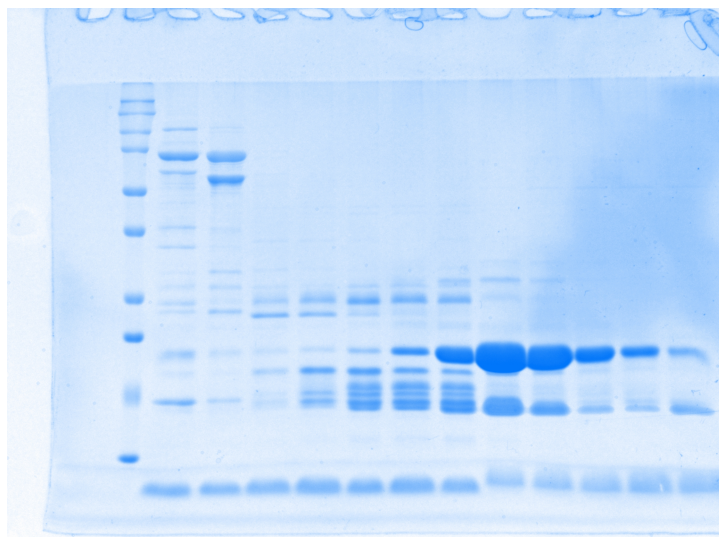

Supplement: Figure 1—figure supplement 1—source data 9. [file elife-89280-fig1-figsupp1-data9.pdf]

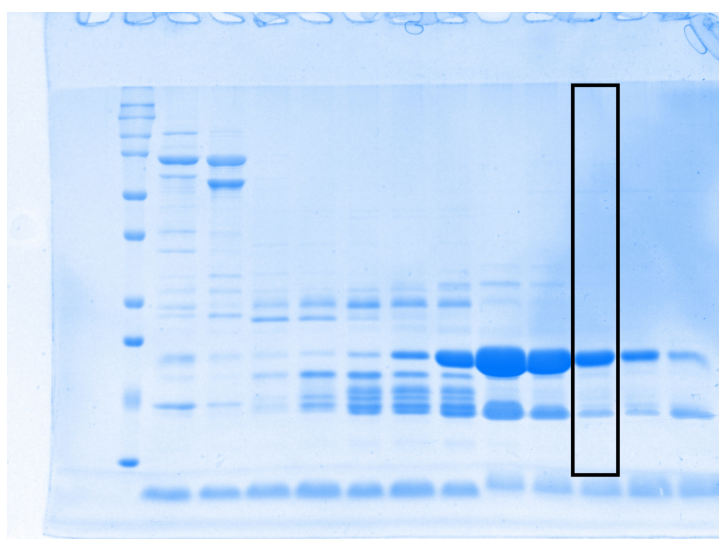

Supplement: Figure 1—figure supplement 1—source data 10. [file elife-89280-fig1-figsupp1-data10.pdf]

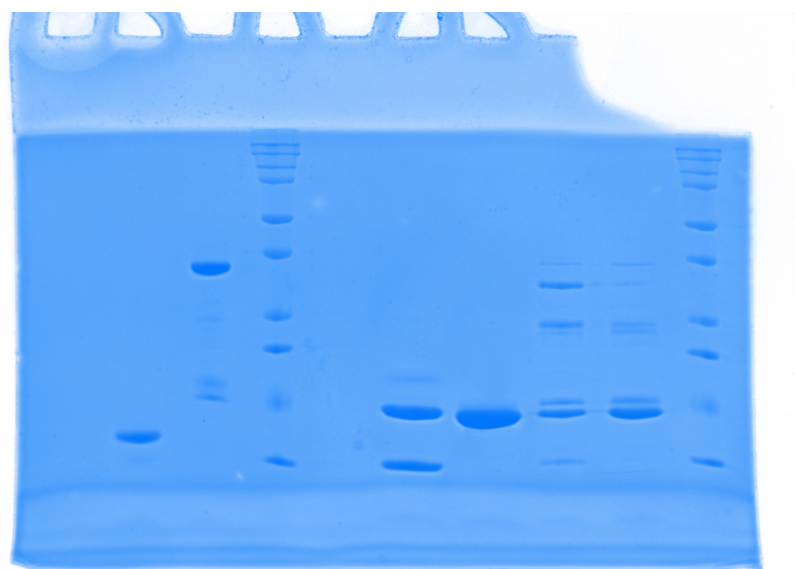

Supplement: Figure 1—figure supplement 1—source data 11. [file elife-89280-fig1-figsupp1-data11.pdf]

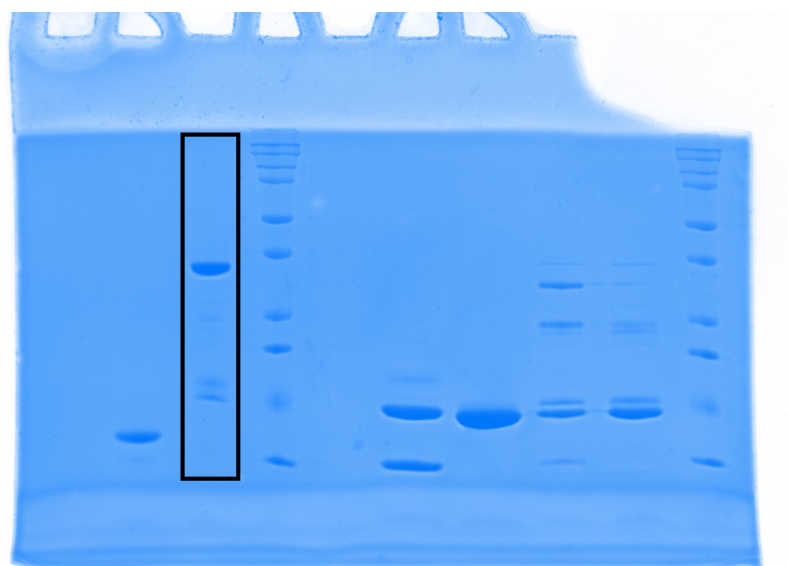

Supplement: Figure 1—figure supplement 1—source data 12. [file elife-89280-fig1-figsupp1-data12.pdf]

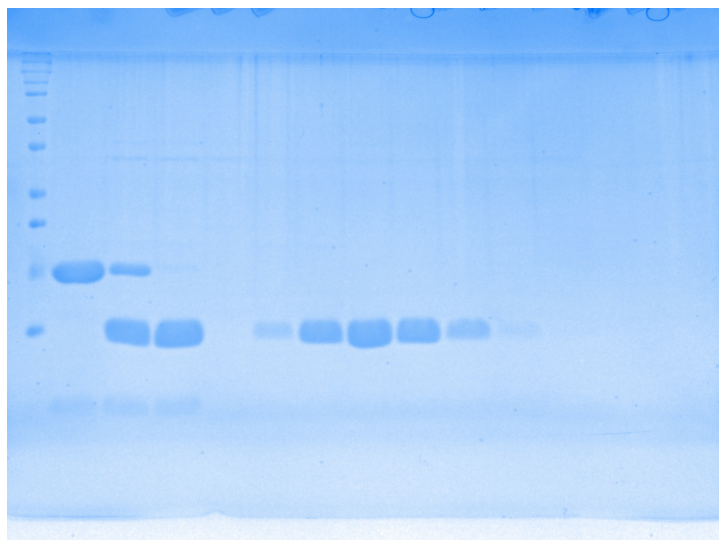

Supplement: Figure 1—figure supplement 1—source data 13. [file elife-89280-fig1-figsupp1-data13.pdf]

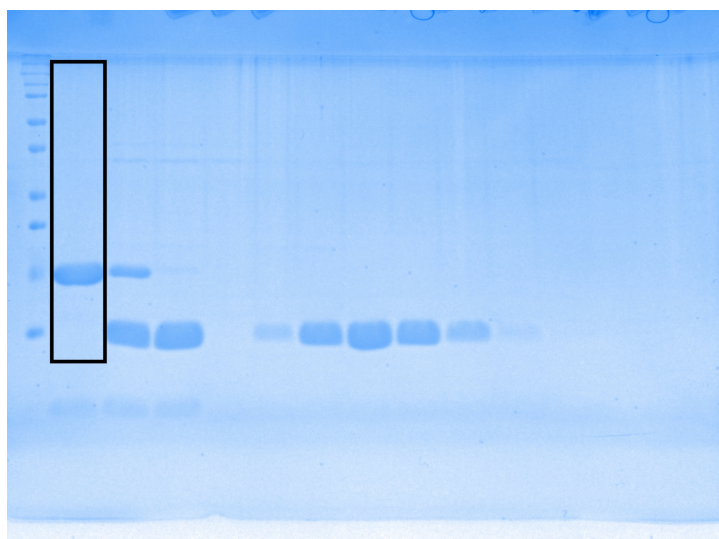

Supplement: Figure 1—figure supplement 1—source data 14. [file elife-89280-fig1-figsupp1-data14.pdf]

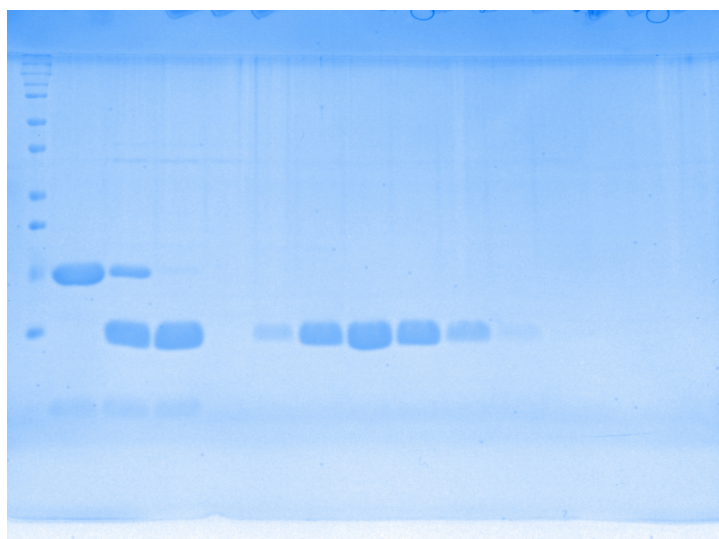

Supplement: Figure 1—figure supplement 1—source data 15. [file elife-89280-fig1-figsupp1-data15.pdf]

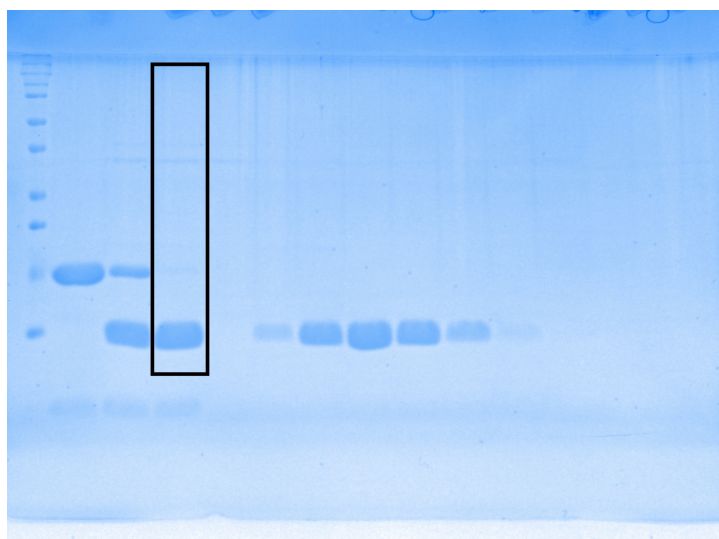

Supplement: Figure 1—figure supplement 1—source data 16. [file elife-89280-fig1-figsupp1-data16.pdf]

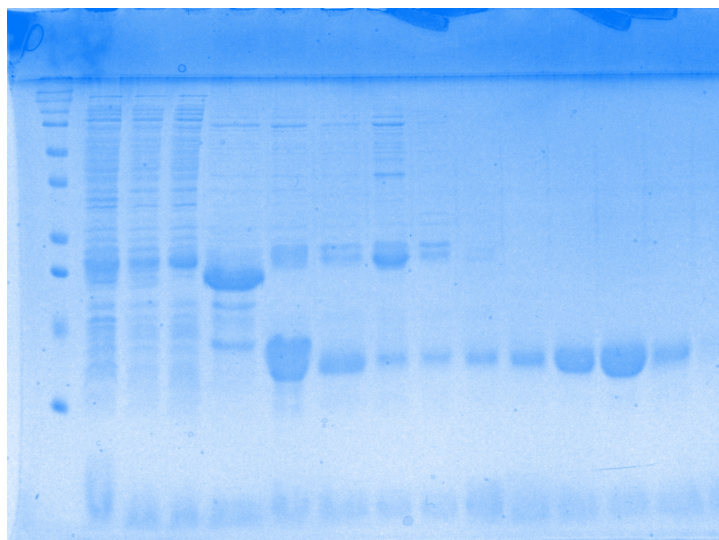

Supplement: Figure 1—figure supplement 1—source data 17. [file elife-89280-fig1-figsupp1-data17.pdf]

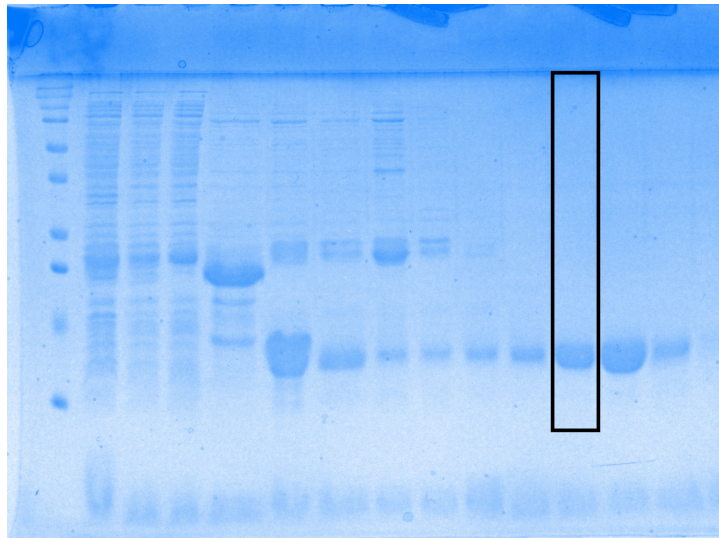

Supplement: Figure 1—figure supplement 1—source data 18. [file elife-89280-fig1-figsupp1-data18.pdf]

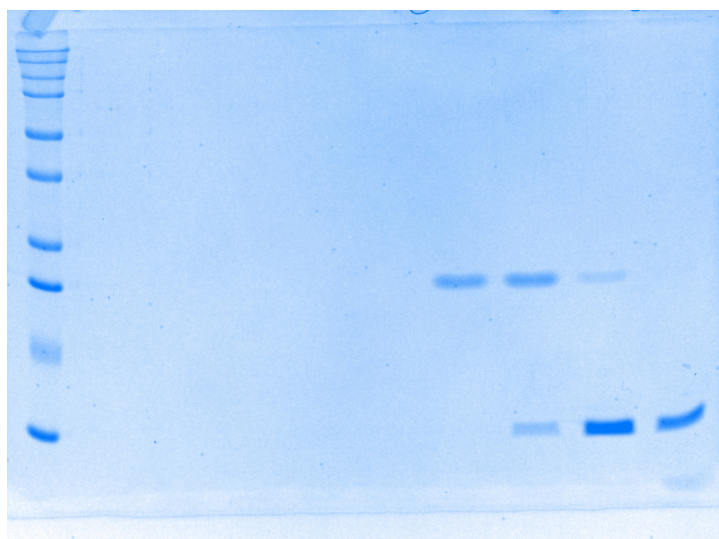

Supplement: Figure 4—source data 1. [file elife-89280-fig4-data1.pdf]

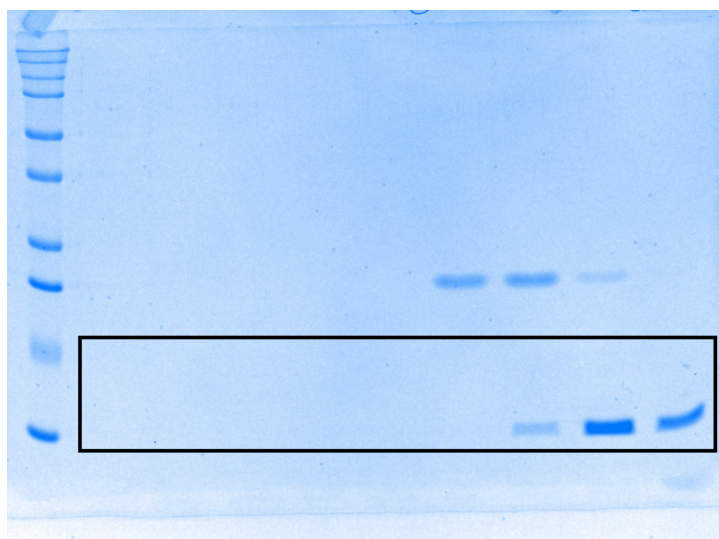

Supplement: Figure 4—source data 2. [file elife-89280-fig4-data2.pdf]

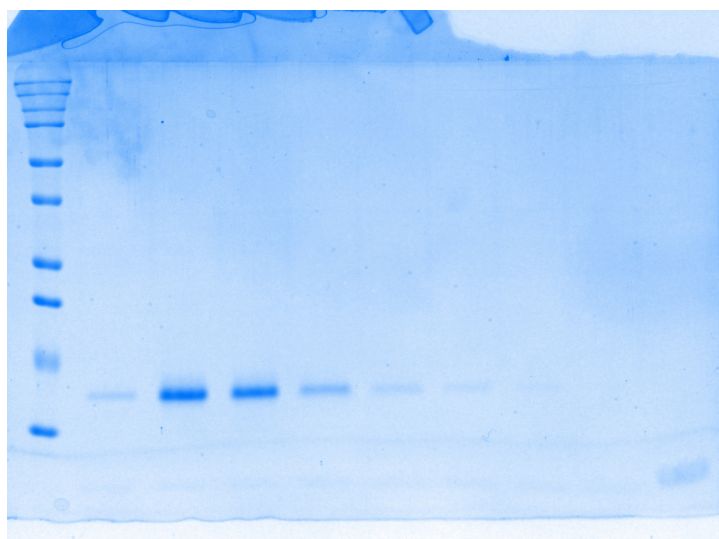

Supplement: Figure 4—source data 3. [file elife-89280-fig4-data3.pdf]

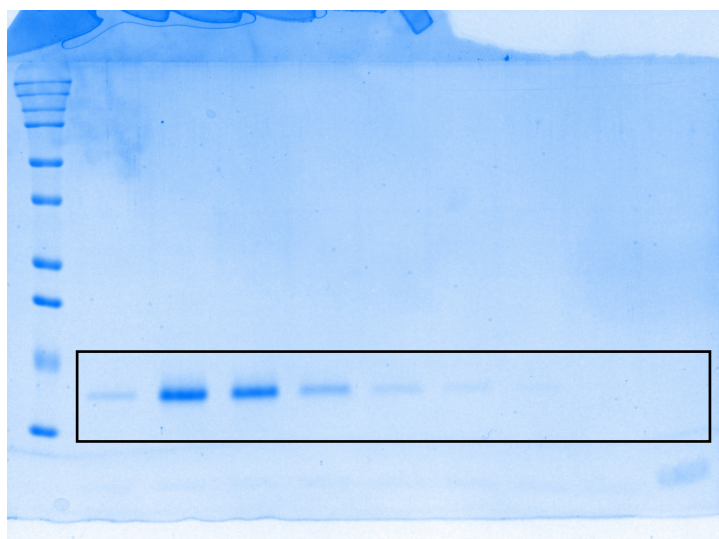

Supplement: Figure 4—source data 4. [file elife-89280-fig4-data4.pdf]

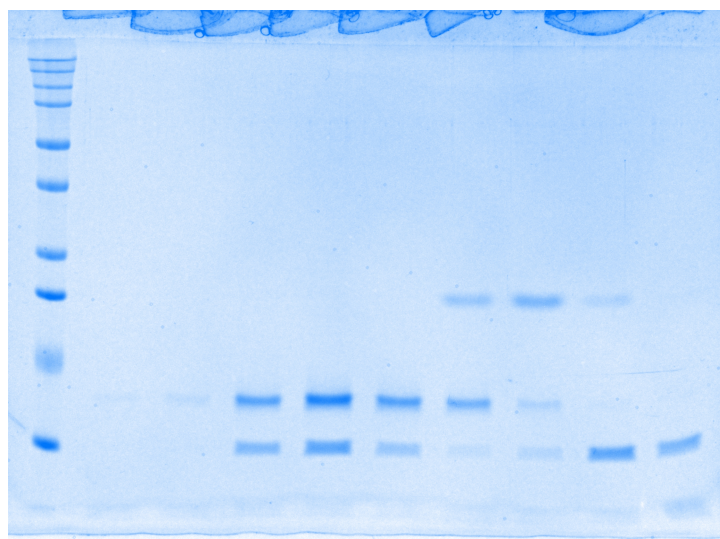

Supplement: Figure 4—source data 5. [file elife-89280-fig4-data5.pdf]

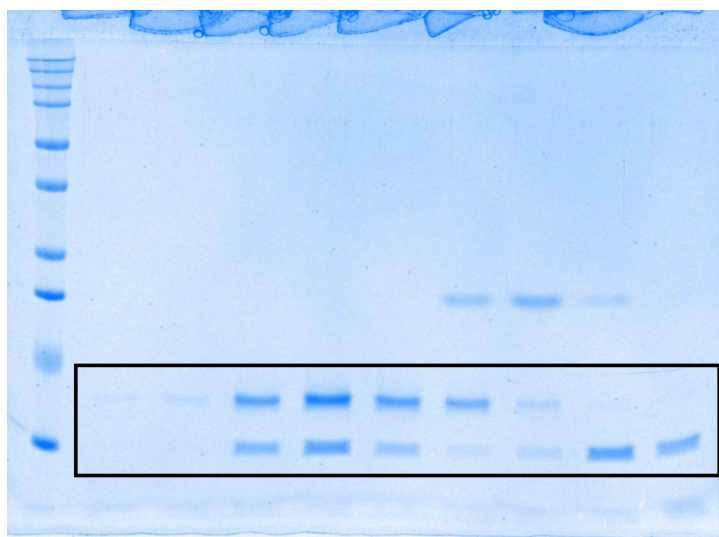

Supplement: Figure 4—source data 6. [file elife-89280-fig4-data6.pdf]

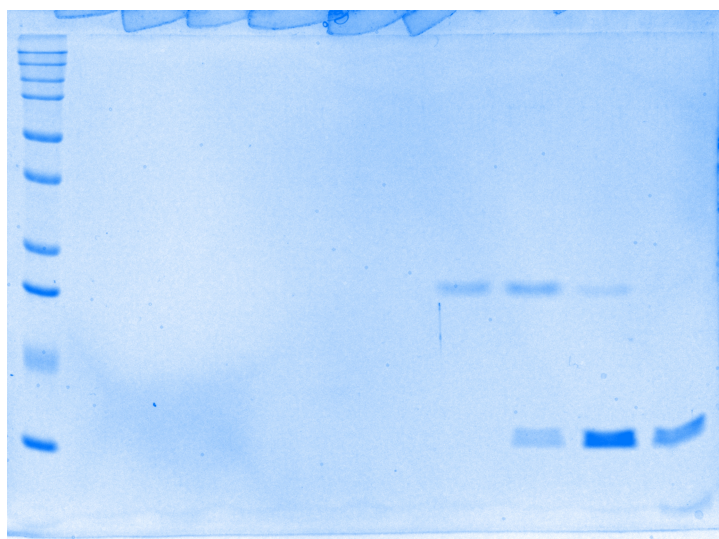

Supplement: Figure 4—source data 7. [file elife-89280-fig4-data7.pdf]

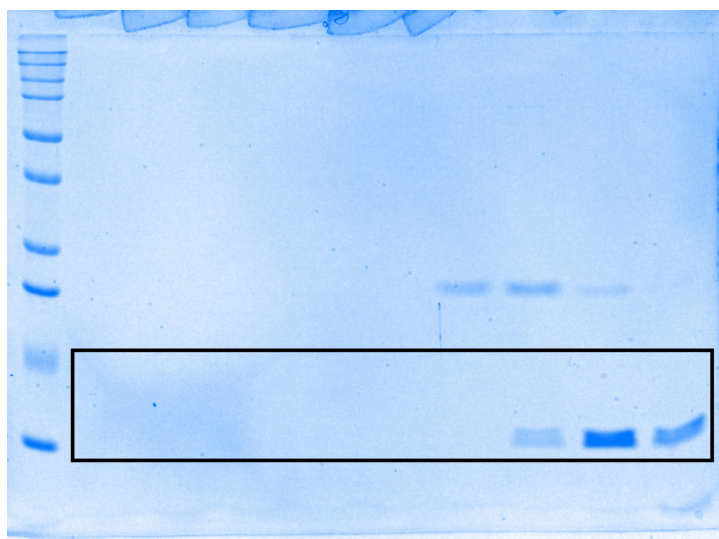

Supplement: Figure 4—source data 8. [file elife-89280-fig4-data8.pdf]

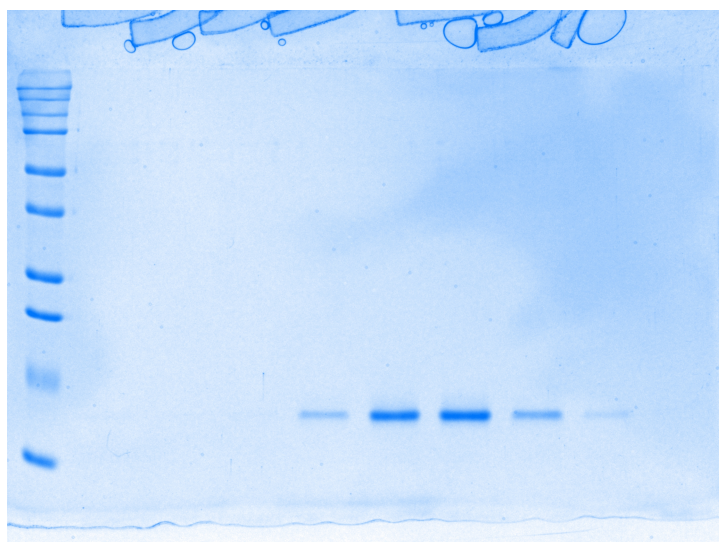

Supplement: Figure 4—source data 9. [file elife-89280-fig4-data9.pdf]

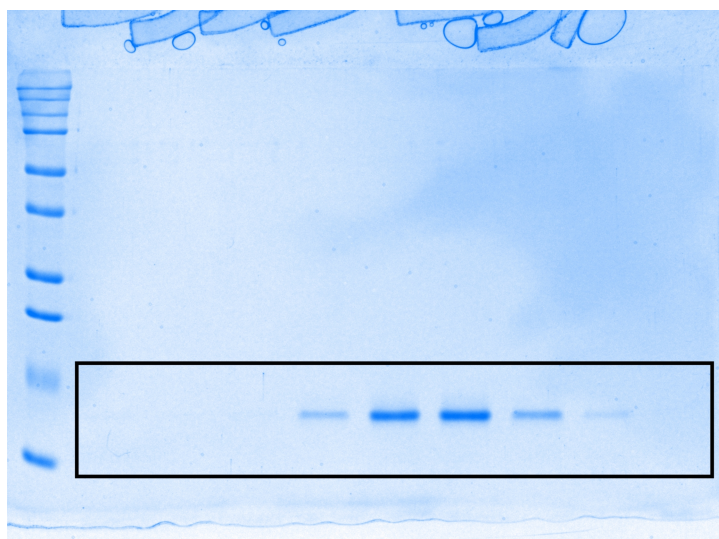

Supplement: Figure 4—source data 10. [file elife-89280-fig4-data10.pdf]

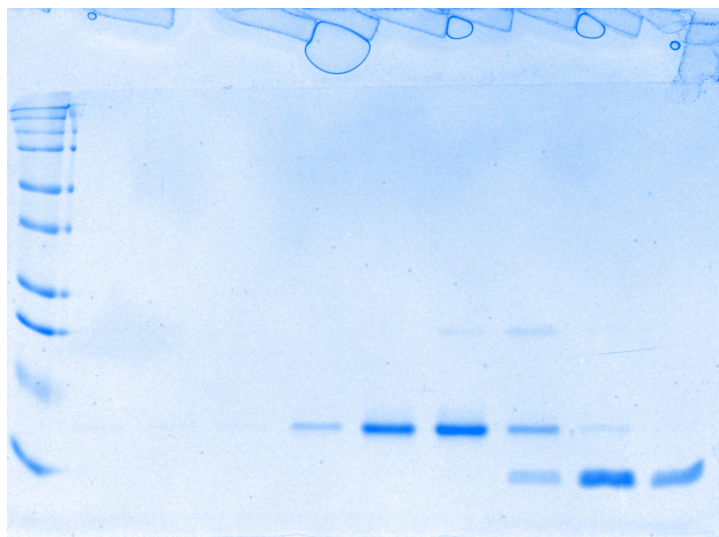

Supplement: Figure 4—source data 11. [file elife-89280-fig4-data11.pdf]

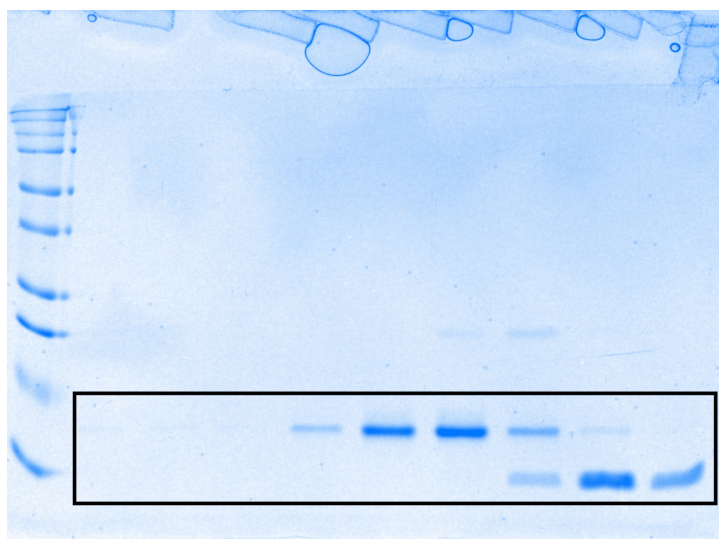

Supplement: Figure 4—source data 12. [file elife-89280-fig4-data12.pdf]

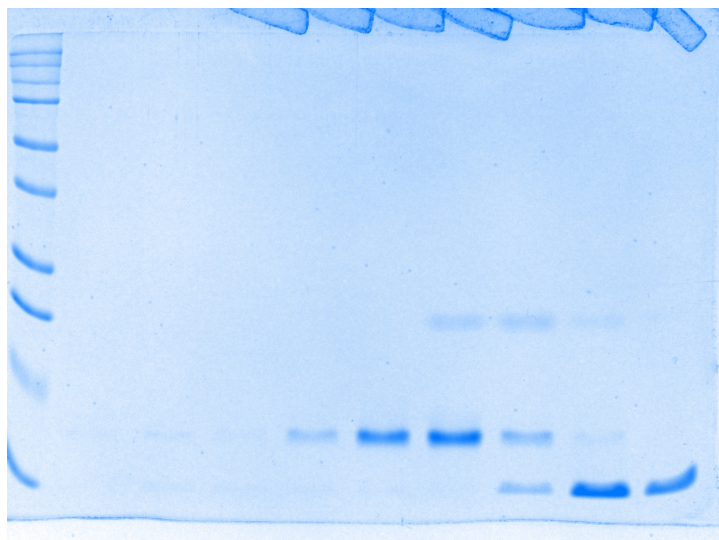

Supplement: Figure 4—figure supplement 1—source data 1. [file elife-89280-fig4-figsupp1-data1.pdf]

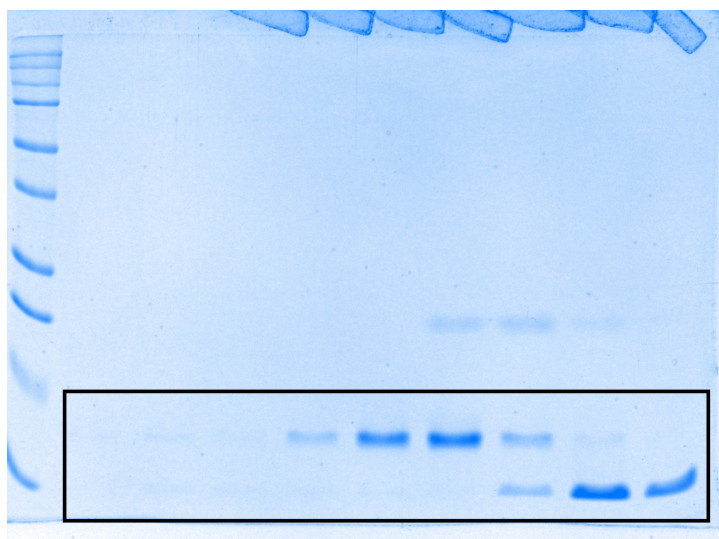

Supplement: Figure 4—figure supplement 1—source data 2. [file elife-89280-fig4-figsupp1-data2.pdf]

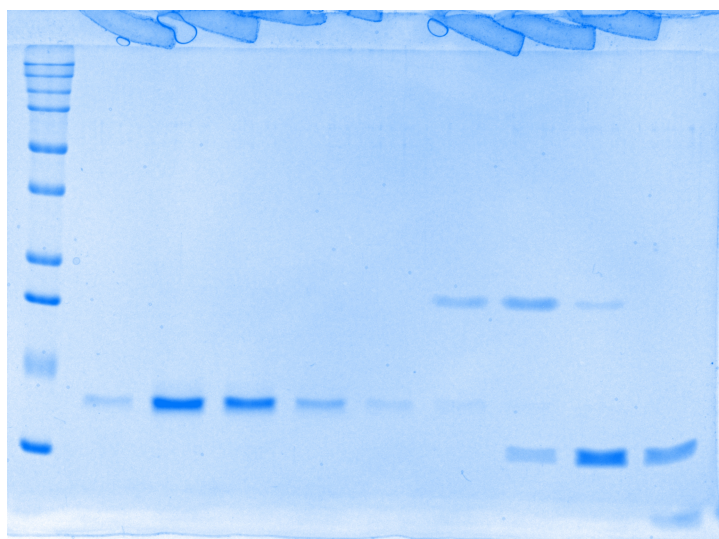

Supplement: Figure 4—figure supplement 1—source data 3. [file elife-89280-fig4-figsupp1-data3.pdf]

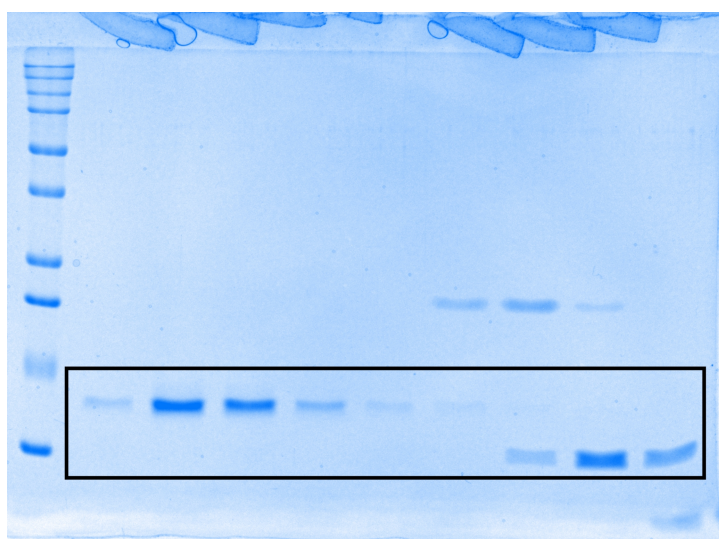

Supplement: Figure 4—figure supplement 1—source data 4. [file elife-89280-fig4-figsupp1-data4.pdf]

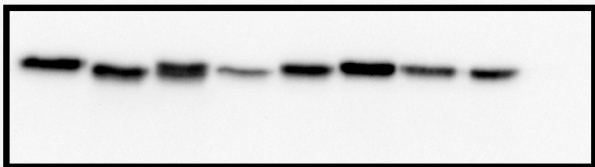

Supplement: Figure 5—figure supplement 1—source data 2. [file elife-89280-fig5-figsupp1-data2.pdf]

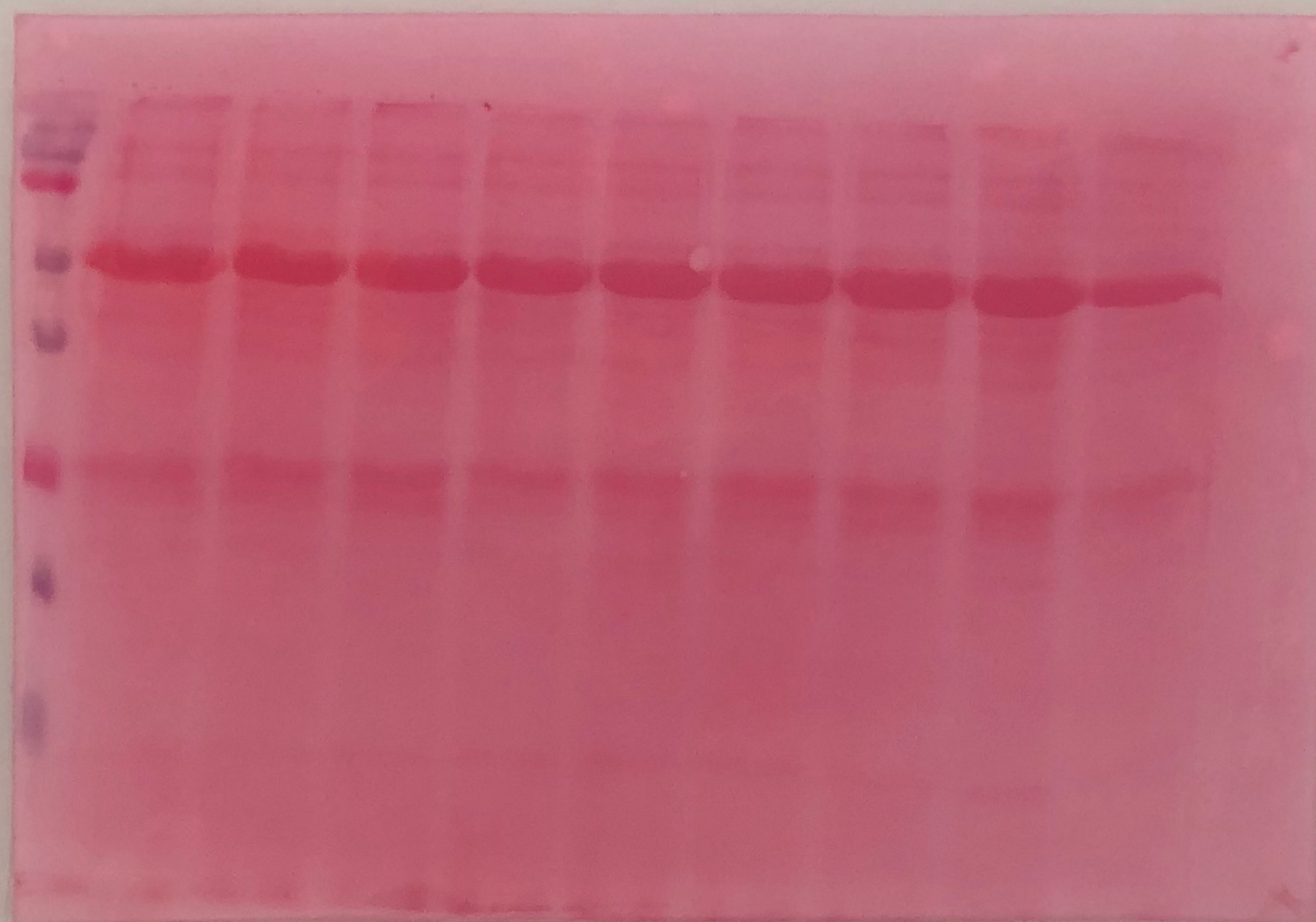

10

Supplement: Figure 5—figure supplement 1—source data 3. [file elife-89280-fig5-figsupp1-data3.pdf]

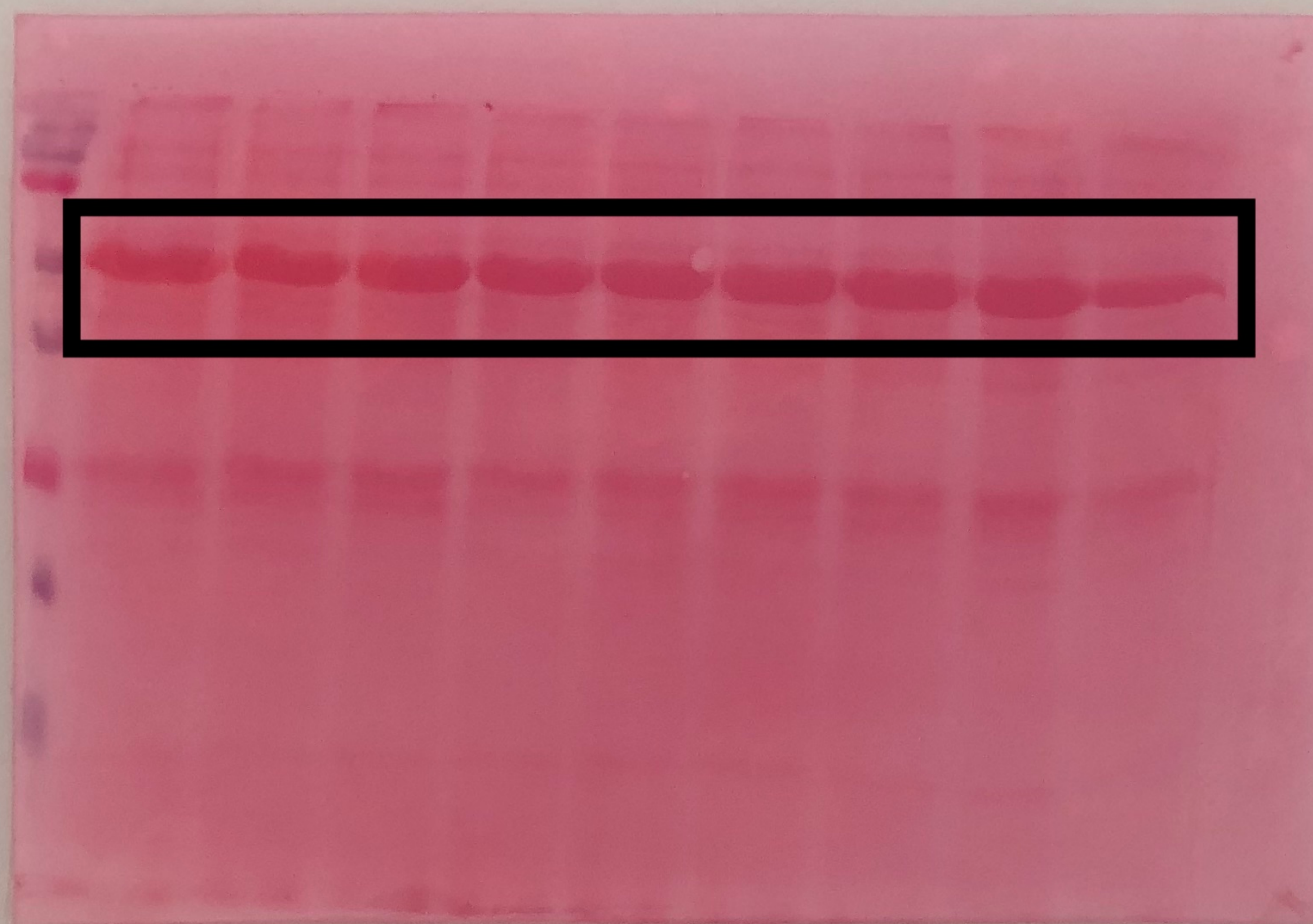

10

Supplement: Figure 5—figure supplement 1—source data 4. [file elife-89280-fig5-figsupp1-data4.pdf]

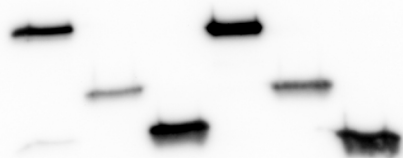

Supplement: Figure 5—figure supplement 1—source data 5. [file elife-89280-fig5-figsupp1-data5.pdf]

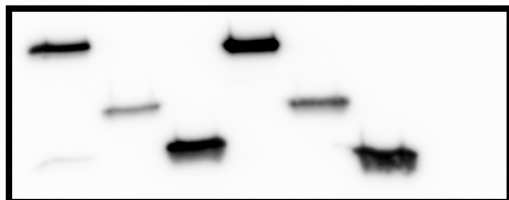

Supplement: Figure 5—figure supplement 1—source data 6. [file elife-89280-fig5-figsupp1-data6.pdf]

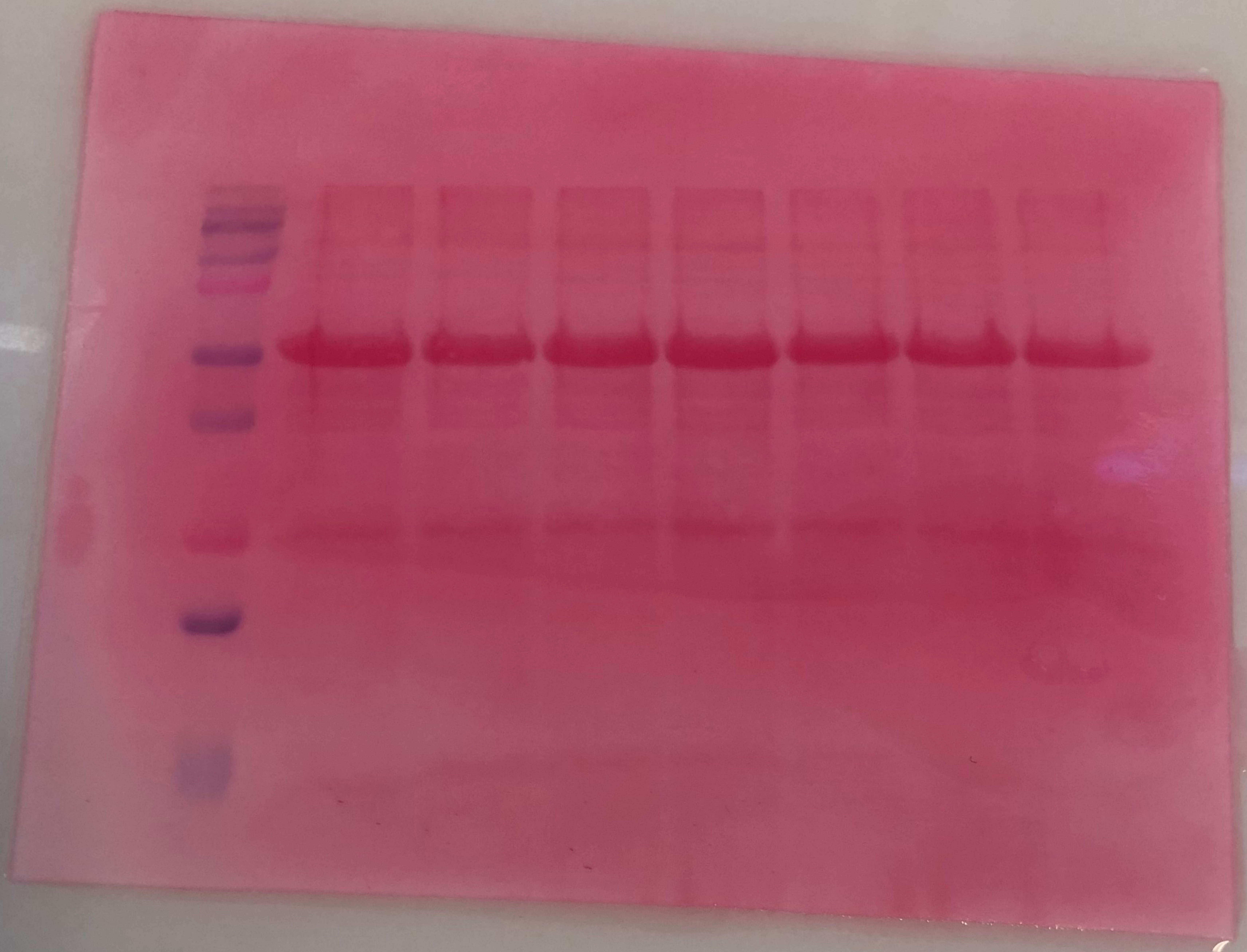

Supplement: Figure 5—figure supplement 1—source data 7. [file elife-89280-fig5-figsupp1-data7.pdf]

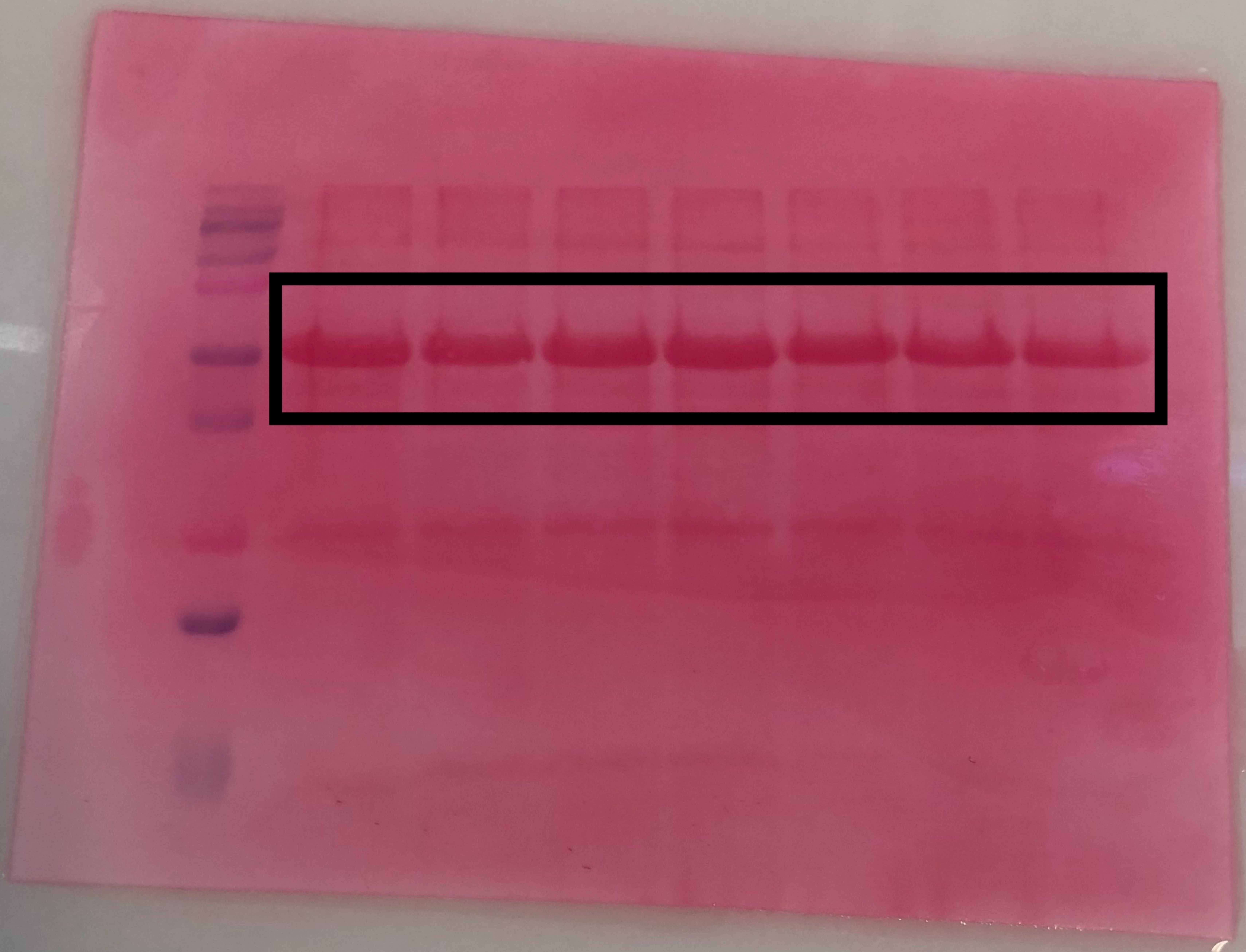

Supplement: Figure 5—figure supplement 1—source data 8. [file elife-89280-fig5-figsupp1-data8.pdf]

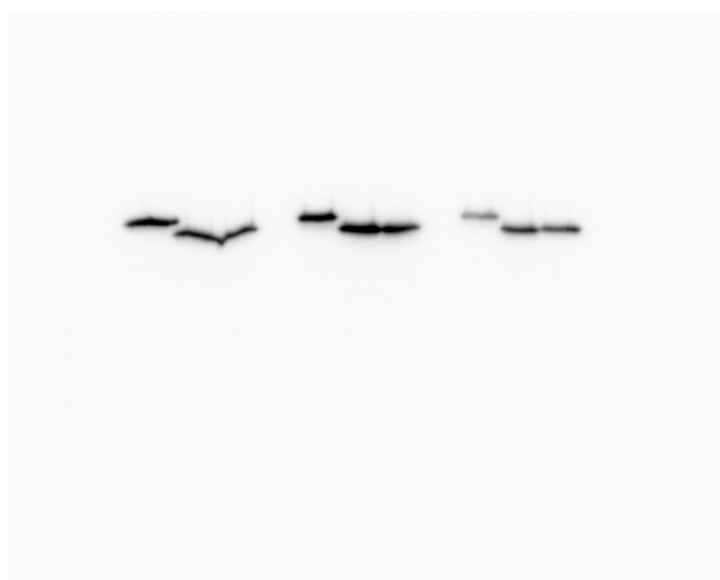

Supplement: Figure 5—figure supplement 1—source data 9. [file elife-89280-fig5-figsupp1-data9.pdf]

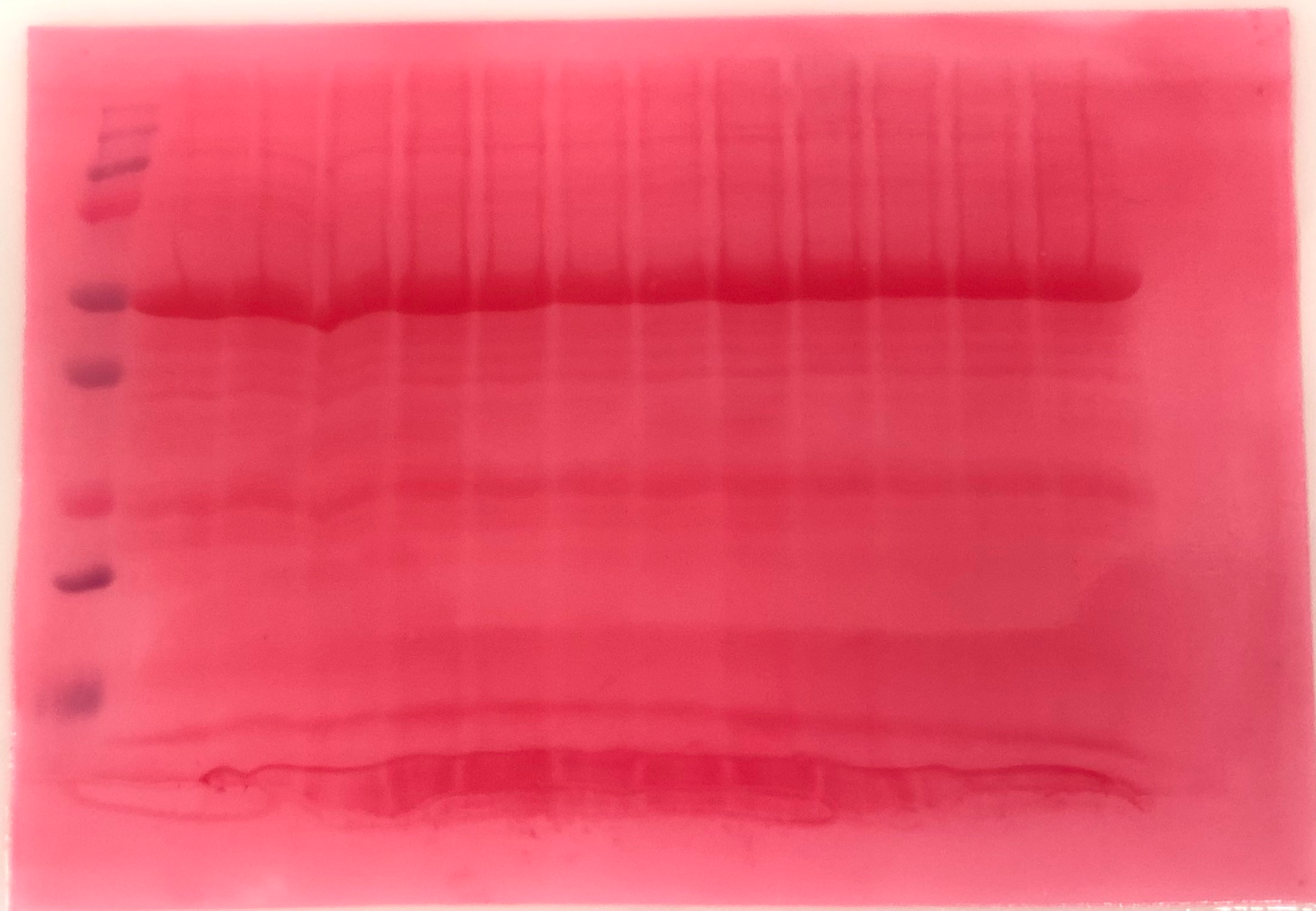

Supplement: Figure 5—figure supplement 1—source data 11. [file elife-89280-fig5-figsupp1-data11.pdf]

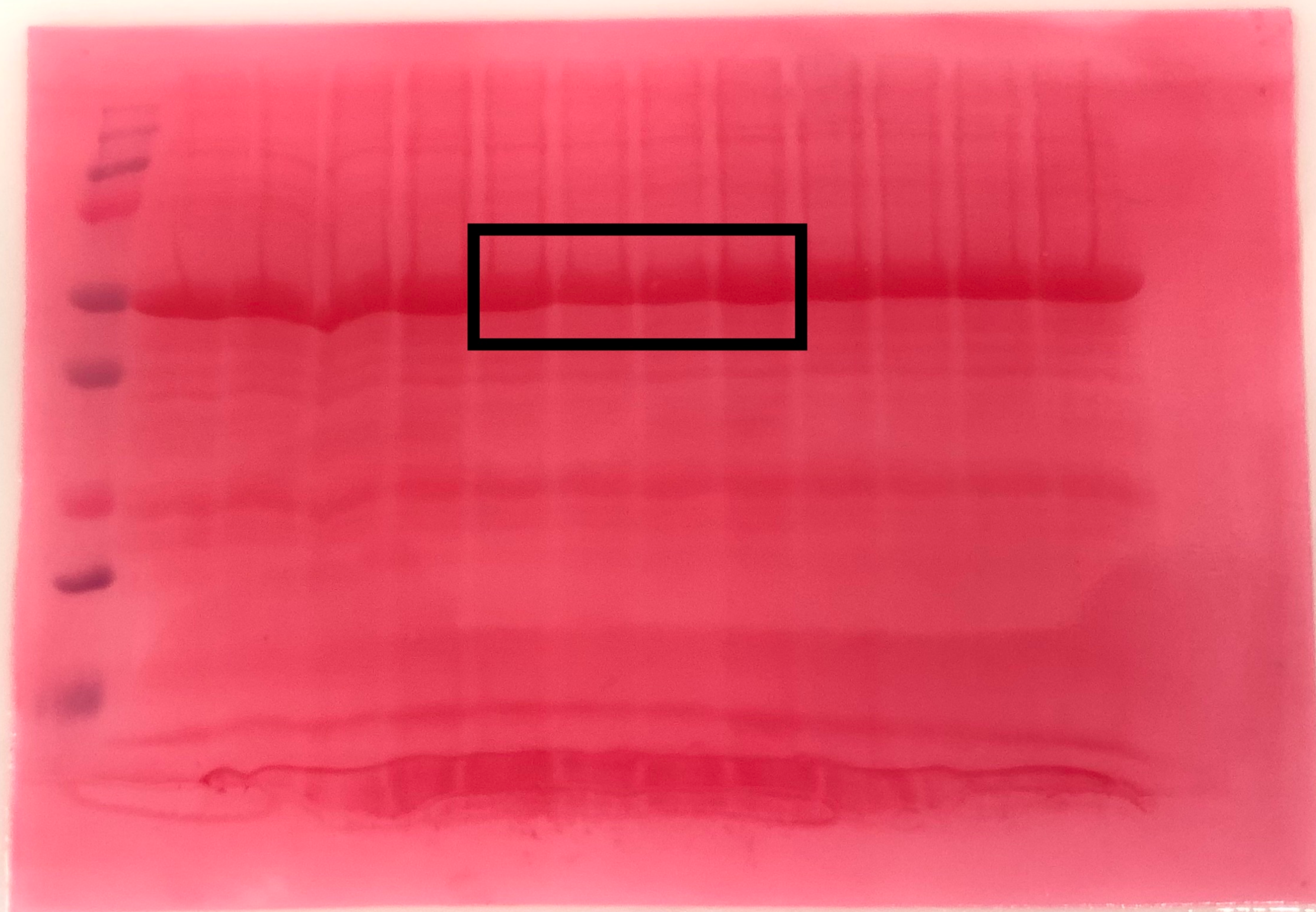

Supplement: Figure 5—figure supplement 1—source data 12. [file elife-89280-fig5-figsupp1-data12.pdf]
