# Supplementary material for: The structural repertoire of Fusarium oxysporum f. sp. lycopersici effectors revealed by experimental and computational studies
Source: eLife. 2024 Feb 27;12:RP89280. doi: 10.7554/eLife.89280 (PMC10942635; doi:10.7554/eLife.89280)
Supplement: Figure 2—figure supplement 3—source data 1. [file elife-89280-fig2-figsupp3-data1.pdf]

<table>
<tr><th></th><th colspan="4">FOLD effectors</th></tr>
<tr><th></th><th>Avr1</th><th>Avr3</th><th>SIX6</th><th>SIX13</th></tr>
<tr><td rowspan="10">Fusarium oxysporum f. sp.</td><td></td><td colspan="3">pisi</td></tr>
<tr><td></td><td colspan="3">melonis</td></tr>
<tr><td></td><td colspan="3">cubense TR4</td></tr>
<tr><td></td><td></td><td colspan="2">vasinfectum</td></tr>
<tr><td>niveum</td><td></td><td colspan="2">niveum</td></tr>
<tr><td></td><td></td><td>passiflorae</td><td></td></tr>
<tr><td></td><td>fragariae</td><td></td><td>fragariae</td></tr>
<tr><td></td><td>conglutinans</td><td></td><td></td></tr>
<tr><td></td><td></td><td></td><td>medicaginis</td></tr>
<tr><td>spinaciae</td><td></td><td></td><td></td></tr>
</table>
