# Supplementary material for: The structural repertoire of Fusarium oxysporum f. sp. lycopersici effectors revealed by experimental and computational studies
Source: eLife. 2024 Feb 27;12:RP89280. doi: 10.7554/eLife.89280 (PMC10942635; doi:10.7554/eLife.89280)
Supplement: Supplementary file 1. [file elife-89280-supp1.docx]

**S1 Table.** X-ray data collection, structure solution and refinement statistics for Avr1, Avr3, SIX6 and SIX8.

|  | **Avr1 Bromide soak - SAD** | **Avr1 Native (7T6A)** | **Avr3 Bromide soak - SAD** | **Avr3 Native (7T69)** | **SIX6 (8EBB)** | **SIX8 (8EB9)** | | |
| --- | --- | --- | --- | --- | --- | --- | --- | --- |
| Detector | Dectris EIGER  2 9M | Dectris EIGER  16M | Dectris EIGER  2 9M | Dectris EIGER  16M | Dectris EIGER  2 9M | Dectris EIGER  16M | | |
| Wavelength (Å) | 0.91946 | 0.95373 | 0.91969 | 0.95373 | 0.95336 | 0.95373 | | |
| Space group | P1 21 1 | P1 21 1 | C2 2 21 | C2 2 21 | P 21 21 2 | P43 2 2 | | |
| Unit cell | 69.87 38.24 80.10  90 103.56 90 | 70.00 40.34  81.30  90 104.54 90 | 54.68 79.93 117.12  90 90 90 | 54.86 80.13 117.37  90 90 90 | 76.303 93.544 60.489  90 90 90 | 51.814 51.814 81.599  90 90 90 | | |
| Average mosaicity (°) ^b^ | 0.00 | 0.08 | 0.00 | 0.06 | 0.06 | 0.05 | | |
| Resolution (Å) | 46.11 - 2.12  (2.18 - 2.12) | 39.35 - 1.65  (1.68 - 1.65) | 45.13 - 2.46  (2.56 - 2.46) | 45.27 - 1.68  (1.71 - 1.68) | 47.40-1.88  (1.92-1.88) | 36.64-1.28  (1.326-1.28) | | |
| Total no. of reflections | 1247183  (94443) | 295538  (14086) | 1426660  (157248) | 280003  (13354) | 243512  (15537) | 381085  (37508) | | |
| No. of unique reflections | 23868  (1868) | 53375  (2645) | 9668  (1079) | 29849  (1458) | 35252  (2205) | 29449  (2869) | | |
| Completeness (%) | 99.8 (98.1) | 99.9 (100) | 99.9 (99.4) | 99.8 (96.8) | 97.8 (96.8) | 99.98 (99.97) | | |
| Multiplicity | 52.3 (50.6) | 5.5 (5.3) | 147.6 (145.7) | 9.4 (9.2) | 6.9 (7.0) | 13 (13) | | |
| Anomalous completeness | 99.5 (96.8) | - | 99.8 (98.7) | - |  |  | | |
| Anomalous multiplicity | 26.0 (25.2) | - | 77.0 (75.3) | - |  |  | | |
| Mean I /s(I) | 24.0 (4.9) | 13.1 (1.5) | 25.1 (5.4) | 14.5 (1.8) | 11.8 (0.9) | 23.71 (1.73) | | |
| Rmerge | 0.157 (0.850) | 0.074 (0.990) | 0.215 (1.258) | 0.072 (0.901) | 0.070 (1.429) | 0.048 (1.35) | | |
| Rmeas ^c^ | 0.158 (0.858) | 0.082 (1.010) | 0.216 (1.262) | 0.076 (0.953) | 0.082 (1.668) | 0.050 (1.41) | | |
| Rpim ^d^ | 0.022 (0.117) | 0.034 (0.470) | 0.018 (0.102) | 0.025 (0.308) | 0.042 (0.857) | 0.014 (0.39) | | |
| CC_1/2_ ^b^ | 0.999 (0.950) | 0.999 (0.490) | 1.0 (0.976) | 0.999 (0.832) | 0.999 (0.571) | 0.999 (0.894) | | |
| Matthews coeff. (Å^3^ Da^-1^) ^e^ | 2.60 | 2.78 | 2.13 | 2.13 | 2.82 | 2.56 | | |
| Resolution range (Å) | - | 39.35 - 1.65 | - | 45.27 - 1.68 | 46.77-1.88 | 36.64-1.28  (1.31-1.28) | | |
| R_work_ (%) ^g^ | - | 16.85 | - | 16.88 | 19.32 | 17.0 | | |
| R_free_ (%) ^h^ | - | 21.10 | - | 21.77 | 22.10 | 19.33 | | |
| No. of non-H atoms |  |  |  |  |  |  | | |
| Total | - | 3647 | - | 1882 | 2998 | 742 | | |
| Macromolecules | - | 3214 | - | 1713 | 2787 | 653 | | |
| Ligand | - | 20 | - | 10 | 20 | 5 | | |
| Water | - | 413 | - | 159 | 191 | 84 | | |
| Average B-factor (Å^2^) | - | 26.06 | - | 28.27 | 40.73 | 23.79 | | |
| RMSD from ideal geometry |  |  |  |  |  |  | | |
| Bond lengths (Å) | - | 0.005 | - | 0.012 | 0.008 | 0.011 | | |
| Bond angles (°) | - | 0.74 | - | 1.18 | 0.93 | 1.21 | | |
| Ramachandran plot, residues in (%)^i^ |  |  |  |  |  |  | | |
| Favoured regions | - | 97.77 | - | 95.57 | 98.58 | 98.85 | | |
| Allowed regions | - | 2.23 | - | 4.43 | 1.42 | 1.15 | | |
| Outlier regions | - | 0.00 | - | 0.00 | 0.00 | 0.00 | | |
| **** | | | | | | |  |  |
