# Supplementary material for: The structural repertoire of Fusarium oxysporum f. sp. lycopersici effectors revealed by experimental and computational studies
Source: eLife. 2024 Feb 27;12:RP89280. doi: 10.7554/eLife.89280 (PMC10942635; doi:10.7554/eLife.89280)
Supplement: Supplementary file 3. [file elife-89280-supp3.docx]

**S3 Table.** Amino acid sequence inputs for AlphaFold2

| **Name** | **Sequence** |
| --- | --- |
| SIX1^96-284^ | EPFGEESRNDRVTQDMLQALHDLCVERFGTGYRAVSGLCYTDRRATRKIECNKPSVRERDRSVTRACPKGQECTTFNAYNFRNRHHQVTFPVCGPRIEVKDRHDIGIHTEWQGTWYPESPKSPGTYDYFAQMAGTLNGYFGYDGVYSDGYKTSSHGYGHSWSCINCPRGKVTITNTYRATWAFGYTSPH |
| SIX2^98-221^ | GSCFSFPTPARGSCMIDYCWRDDNGVIYSRGITITGSNGASNPTSMRSNDPANLSLNSVFNDGYNGWFPHGHACSNSDTQIYTNHRLLQGVNGVAYVDHVRCENCNFRNVNCLSDVLKNNLIAYSNGVASQSRCT |
| SIX3^36-163^ | LPVEDADSSVGQLQGRGNPYCVFPGRPTSSTSFTTSFSTEPLGYARMLHRDPPYERAGNSGLNHRIYERSRVGGLRTVIDVAPPDGHQAIANYEIEVRRIPVATPNAAGDCFHTARLSTGSRGPATISWDADASYTYYLTISED |
| SIX4^59-242^ | SAHTESVCVHAGTATGADLHWLNAICTGKSTYTVNCAPAGNKNAGSTHTGTCPAGQDCFQLEQVGNFWGDREPDATCSPSNTVFDAVDDKEATHVNGKVVTRAGKPGIGRKLIRLKAQVYRRDGHYGQTSRMGFFRNGKEVYHIDNVASMEPTWNFDPSSDQSFSFFFTPGPNAFRIQGTLNLA |
| SIX5^18-119^ | RDHQYCACQSGSGDSIDIDATTQLQNDNSKSYLWAQTSPAYWFADRHKPGPRFAGIYLKAANGKIDGDTFYNLCINNGGADSTCFDCSKSHQVRNVIYCDAA |
| SIX6^58-225^ | DTLPVSTCPAGQKYDRSVCYKADKIRSFCVANPRSNREKITDTPCQPREICVQRNLSNGKSFAKCIPIVDLVEWKTSANGNKEGCTTTSVNPAGYHHLGTIVYDINKNPIEVDKISYFGEPGNVNEGIGGSTSYFSSDNFQFSKSRYMKTCIFSGGYGNLNAYTWSWE |
| SIX7^49-220^ | EVTFDITQNVNTFTSAASTPWTEGVGLSNIRYQWRAYYSTRQRTTFVEVRVFGTAEAQVVLLPDAPGTSRYRAIDSNVFRPNEEVTGGGLAGWGQVTTVCLQTWGRRGDITYRLRIQS |
| SIX8^50-141^ | DTSGILLACITGAGSAFQAYAGCYLTAFRNDPRTLTLRMDKTRGERISNVLVILSGGALSHAVEEVVQIAPGAVRNLATLGASTVQFLHNFR |
| SIX9^19-114^ | QTTQVGCRALDTKNDGLLTELLLNPSARGAADPDLRYGFWDAKWRKCCNKYKECDKYYTFSYNHPYPWAYRQRRGTIRGQQFDFACVNWRTGACK |
| SIX10^15-149^ | IPDSGVSTGTKDLSKRDDAYIFDVTFRVGPAGANVAPFSGSVYVQDGLTPLVRSGSGSSISDRGYNAFRGIVYFTFTHGYNQYSASTRFGVYVDTGLIVDSNGRPIYGTAPRKACIDYSPHGPTDVCSVTITRSK |
| SIX11^19-110^ | INICCSSFAGHTCTKDQYNNHRQNVILNQIIDKDGVNCVRKGAGPGRWTRKGDWSEWYDCQQWNGPEQHQIEVGECTLFCVTPSGILNRPCI |
| SIX12^27-127^ | SSCLSVGPKGISNQNACVCGGQCVMKDLVVARRKVCCEYTVQIQGGWPVLAQSRCVYGSTGANGGSCSGDNVSLAWWLNYEPEVKSTDPTCIFAKPKLCHS |
| SIX13^78-293^ | QDDEHPNGPCPRGGRLYVDSDEDSSCNAKWGTQTHNDVKTFGSTGSVCAGTFRRITCACCYTMHPITDNNVPRMDGIYCPKWEVCKQEPERWSKWGNRVSHTSCVQAKKLTEILIATKKVVKEYCTPKRWLPSAGKGKNAKFHTWAYNYSTGQLTTLKWMYLKLDGQYVKSAPGISEWGLTYSVNEHNAIELCGYPSDDMQRNSIDAELQWEATVQ |
| SIX14^18-88^ | QRILGCRMPNGSLNPSPNICNQAGGSFRSGSRGCCTRNTRDGPVVTESRFISGCNKNGGFVSSKEILATSC |
| PSL1^18-111^ (FOXGR_025399) | EDWDRCRCMKYPETGTPNDCATIKACGSGKHRAISISEEKGDIWCEKTDVAISGPEFYRTCYGLLQDPKPNSEADSCCTRWVDGVVVQSDGCFK |
| FOXG_11033^24-226^ | APEAAPGYTTYGDYKGAGENLPSYPSYGSYGAKPKPKPKPAPAPKKYTNYGSYNYKKYSSYGHYKREAEPEAAPEAAPEAAPEAEAAPGYTTYGDYKGAGENLPSYPSYGSYGSKPKPKPKPAPAPKKYTNYGSYNYKKYSSYGHYKREAEPEAAPEAEAEPETYSKYGSYPKKYTHYGSYNYKKYSSYGTYKRAKEFINSLF |
| FOXG_05755^56-157^ | NGVPDPGNFAASCHGLQVISDDLDLTGKPDFERCNDTAYEARQYFSGEYTTVEVRRTDYPDLGKEVQISATANYTSTNDNIVNGHLKFGDFQTKFISTPIEG |
| FOXG_18699^21-96^ | CKRTCSASNDAGTTCSYSCTQVCSSISAKQARDTFLAALQSGGNSCSAVGTSGVSCRKTAKFGSCYDHHWSCGSGC |
| FOXG_04863^106-300^ | MYDSSDDKGGLSDITRNAWSKFCNSPYGNNGGVTTRFILDGQWGAVGRLSGWSMRDALIHSMWQTADGIGKKNGYTVYNGCYGFTWQESKPGKANSACGGRSGKACPYNDDCPLAGMECTGLKWGTWMPSIIRMNVYNRDGSLRADAYQARISSQAVGSGGCSKAQTISAYVADFIPIVGPYFATGIRINCLYQS |
| FOXG_04805^39-132^ | QNGQNGGRPVPSGECCVANTSLKQDACTASNGQAGRCVPGGNNCGGRLSCVAQANLQCDANVIERGKDLCRAKAANGLFDGGNIIQNLSQAKVN |
| FOXG_02829^18-149^ | APSSPSDIQARSCVCKKVGDDWICTGTKCYDKVKRDLVPRQCSCHKIGDEWLCGGPKCPRDLPEENKLAKRQCSCHKVGDEWLCGGPKCPRSLPAEESGLEKRQCSCKKVAGEWICSGRKCPRDLSHLMGEE |
| FOXG_16600^17-164^ | SPISKRAVFSQTTYDDLSISGGTAGNAQQEALQKLGGLPTDLSTVEKSDLDFLNSVNQIANDAEDEAFNPAIDAASGEAADALQRGKIKNKVLKLTATILKLQAQQAQGEDVADKLAEENKKLQNNISQDKDEAGKASTFLAFDATTS |
| FOXG_14684^38-168^ | DGTCPRPMCTTPASQGPKDPPACGDSYAACKFDQFPCDEYFSPKVTDTHHCYCILANKKAMDAYCQERGFKSGTNPWKYYYAVECHGAVSNQVCNKDCRDQGRGKGRIDKAHPNGACACDKPNPPYDTCKP |
| FOXGR_007323^18-86 *^ | SLVRRVDVNVPAMTNADGVVVPFDTAGVVQPAKKRDLEQKKRDLAQRKRHISRKRRAVSQEKQKQQQKQ |
| FOXGR_025639^18-61 *^ | APVVRGPGGRLVQEGAGCTLVQGRSVCDDGFGNTFFEDDPFSSK |
| PSL2^18-106 *^ (FOXGR_015322) | EDWDQCRCMKYPSTGTPNDCATIKACGSGKHRAISIYKNGDIWCEKTDVAINGPEFYRTCYDLLQDPKPNSEADSCCIKGDRASDGCFK |
| FOXGR_015533^19-114 *^ | QTCAIAPDPQRNADAFSATSHSGNIDIAFRDHVVFARPSAGTATGVLRLSNGDSYRKIYRIAGPNNVAQFYWLDASSQCKTNLAITQMTNAAWYKE |
| FOXGR_015522^19-79 *^ (SIX15) | TIYCRDVSPPRDTRSWCKTNTPAWQGCQRFCSEHCRSTPRDYPDGCMYHLQVGGDYDCFCK |
| * Effector candidates identified in the reannotation of the *Fol* genome by Sun et al. (2022) and not predicted in the original genome annotation by Ma et al. (2010). | |
