# Supplementary material for: The structural repertoire of Fusarium oxysporum f. sp. lycopersici effectors revealed by experimental and computational studies
Source: eLife. 2024 Feb 27;12:RP89280. doi: 10.7554/eLife.89280 (PMC10942635; doi:10.7554/eLife.89280)
Supplement: Supplementary file 4. [file elife-89280-supp4.docx]

**S4 Table.** DNA sequences of synthesised gene fragments used in this study.

| **Name** | **Sequence (5’ - 3’)** |
| --- | --- |
| SIX6* | TAGGTCTCCAATGGGTCCCTTAGCCCAAACAGAATCCGAGTCGGCAGACGTCGCTGAACATACAATCAATTATATCGACATTGCCCCTGAAGAATTTGAACCGCCCAAAGCTAATTTGTCATCTCTGGTGAGTCGTGACACGCTTCCTGTCAGTACCTGTCCTGCGGGTCAGAAATACGATCGTTCCGTGTGTTACAAGGCAGACAAAATTCGTAGCTTTTGTGTCGCAAACCCTCGTAGCAACCGTGAGAAGATTACCGACACACCGTGTCAGCCCCGTGAAATCTGTGTGCAACGCAATCTTTCCAACGGCAAGAGTTTCGCTAAGTGTATCCCCATTGTAGACCTGGTGGAATGGAAGACATCCGCAAATGGGAATAAAGAAGGCTGTACTACAACGTCCGTGAATCCGGCTGGGTACCATCACCTTGGTACTATTGTTTACGATATCAATAAGAATCCTATCGAAGTTGATAAAATCTCGTACTTCGGCGAGCCGGGAAATGTAAACGAGGGCATTGGTGGCAGCACAAGCTATTTTAGTAGTGACAACTTTCAATTTTCTAAGTCCCGCTACATGAAAACTTGTATTTTCAGTGGTGGGTACGGGAATCTTAACGCCTATACGTGGAGCTGGGAATCTTGGAGACCGT |
| SIX6-TEV* | TAGGTCTCCAATGGGTCCCTTAGCCCAAACAGAATCCGAGTCGGCAGACGTCGCTGAACATACAATCAATTATATCGACATTGCCCCTGAAGAATTTGAACCGCCCAAAGCTAATTTGTCATCTCTGGTGGAAAATCTTTACTTCCAGTCCGACACGCTTCCTGTCAGTACCTGTCCTGCGGGTCAGAAATACGATCGTTCCGTGTGTTACAAGGCAGACAAAATTCGTAGCTTTTGTGTCGCAAACCCTCGTAGCAACCGTGAGAAGATTACCGACACACCGTGTCAGCCCCGTGAAATCTGTGTGCAACGCAATCTTTCCAACGGCAAGAGTTTCGCTAAGTGTATCCCCATTGTAGACCTGGTGGAATGGAAGACATCCGCAAATGGGAATAAAGAAGGCTGTACTACAACGTCCGTGAATCCGGCTGGGTACCATCACCTTGGTACTATTGTTTACGATATCAATAAGAATCCTATCGAAGTTGATAAAATCTCGTACTTCGGCGAGCCGGGAAATGTAAACGAGGGCATTGGTGGCAGCACAAGCTATTTTAGTAGTGACAACTTTCAATTTTCTAAGTCCCGCTACATGAAAACTTGTATTTTCAGTGGTGGGTACGGGAATCTTAACGCCTATACGTGGAGCTGGGAATCTTGGAGACCGT |
| Avr1Thrombin* | TAGGTCTCCAATGTTGCCTAAAGGAGAGGAGGGTGACATTATTGGTACTTTCAATTTCTCGTCCAGCGACAGCCAACCCCTTAAAATCCACTGGGTCGATACGCCGGACTCATCTGGGAGCAATCTTGTTCCCCGTTCCGCTCACACGGAGAGTGTATGCGTTCACGCCGGGACCGCTACAGGTGCTGATCTGCATTGGTTGAATGCGATCTGCACCGGGAAGTCTACATACACAGTGAATTGCGCCCCGGCAGGCAACAAGAATGCTGGGTCTACGCACACAGGAACATGTCCGGCAGGTCAGGACTGTTTCCAATTAGAGCAGGTCGGAAACTTTTGGGGGGACCGTGAGCCAGATGCTACCTGTAGCCCGTCCAATACGGTATTTGACGCCGTAGATGACAAGGAAGCTACGCATGTAAACGGCAAAGTTGTTACACGCGCGGGGAAGCCGGGCATTGGGCGCAAGCTTATTCGTCTTAAGGCTCAGGTCTATCGTCGTGATGGTCACTATGGTCAGACCTCGCGCATGGGATTCTTTCGTAACGGCAAAGAGGTTTACCATATCGACAACGTTGCCTCGATGGAACCCACTTGGAATTTTGACCCATCGAGTGACCAATCCTTTAGCTTCTTTTTCACACCGGGACCCAACGCTTTCCGTATTCAAGGAACGCTTAATCTGGCCTCTTGGAGACCGT |
| SIX8Thrombin* | TAGGTCTCCAATGACCCCGATTGACAAATCGTTAGATCAAGCGGCAACTATCGAAGAAACTGTCCACCAACCTCACTCCCATGATGAGCGTGCCTTAGTTCCACGTGGCAGCGATACGAGTGGGATTTTGCTGGCGTGTATTACCGGCGCAGGATCTGCGTTTCAGGCGTACGCTGGATGCTACTTAACAGCTTTCCGTAATGACCCTCGCACTTTAACTTTGCGTATGGATAAAACCCGTGGAGAACGTATTTCCAATGTTCTGGTTATCTTGTCAGGGGGTGCATTGAGTCACGCCGTGGAAGAAGTAGTACAGATTGCGCCTGGAGCGGTCCGCAATTTGGCAACATTAGGAGCTTCGACTGTCCAATTCCTTCATAATTTTCGTTCTTGGAGACCGT |
| PSL1* | TAGGTCTCCAATGGAAGATTGGGATCGTTGTCGTTGCATGAAATACCCCGAAACTGGGACGCCGAATGACTGTGCCACCATTAAAGCGTGCGGTTCTGGCAAGCACCGTGCCATCTCTATTTCGGAGGAGAAAGGAGACATTTGGTGCGAAAAGACTGATGTAGCAATCAGTGGACCGGAGTTCTACCGCACTTGCTATGGGTTGTTGCAGGACCCGAAGCCTAACTCGGAGGCAGATTCTTGTTGTACTCGTTGGGTAGACGGCGTTGTGGTACAATCTGACGGGTGCTTCAAATCTTGGAGACCGT |
| SIX13* | TAGGTCTCCAATGGAACTTGAAGTTTCCGATTTAAGTGATCAACCGCCGTCAGTGGAGAATACTTACCGCGACCAAGCGTTCAATGAGGAGGAGTTGTTAAAGGTCGTGGACGAATTATCCGTGGAGCGTACCGAACACACCGAACGCGCGCTTGTGAGTGAAGCGGCAGTCCAAAAGCGCCAGGACGACGAACATCCAAACGGTCCTTGTCCTCGCGGAGGACGTTTGTACGTGGATTCTGACGAAGACTCTTCTTGTAATGCGAAGTGGGGAACACAGACACATAACGATGTTAAGACTTTTGGCTCCACCGGATCAGTCTGTGCCGGTACTTTTCGCCGCATTACGTGTGCTTGTTGTTACACGATGCATCCTATCACGGACAACAACGTTCCACGCATGGATGGTATTTACTGCCCTAAGTGGGAGGTGTGCAAACAAGAGCCTGAACGCTGGTCTAAGTGGGGCAATCGTGTAAGCCACACATCTTGCGTACAGGCGAAGAAGCTGACCGAAATCCTGATCGCTACTAAGAAAGTGGTGAAGGAATACTGCACCCCAAAACGTTGGTTACCATCTGCGGGAAAAGGCAAGAACGCGAAATTCCACACGTGGGCGTACAATTATTCGACGGGTCAGCTTACAACCTTGAAGTGGATGTATTTGAAGTTGGACGGTCAGTATGTCAAGAGTGCGCCTGGGATCAGCGAGTGGGGGCTTACCTATTCAGTCAATGAACATAACGCGATTGAGTTATGTGGTTACCCAAGCGATGATATGCAACGCAATTCGATCGATGCTGAGCTTCAATGGGAGGCTACCGTACAGTCTTGGAGACCGT |
| SIX8_C58S Thrombin* | TAGGTCTCCAATGACCCCGATTGACAAATCGTTAGATCAAGCGGCAACTATCGAAGAAACTGTCCACCAACCTCACTCCCATGATGAGCGTGCCTTAGTTCCACGTGGCAGCGATACGAGTGGGATTTTGCTGGCGTCTATTACCGGCGCAGGATCTGCGTTTCAGGCGTACGCTGGATGCTACTTAACAGCTTTCCGTAATGACCCTCGCACTTTAACTTTGCGTATGGATAAAACCCGTGGAGAACGTATTTCCAATGTTCTGGTTATCTTGTCAGGGGGTGCATTGAGTCACGCCGTGGAAGAAGTAGTACAGATTGCGCCTGGAGCGGTCCGCAATTTGGCAACATTAGGAGCTTCGACTGTCCAATTCCTTCATAATTTTCGTTCTTGGAGACCGT |
| PSL1_C37S* | TAGGTCTCCAATGGAAGATTGGGATCGTTGTCGTTGCATGAAATACCCCGAAACTGGGACGCCGAATGACTCTGCCACCATTAAAGCGTGCGGTTCTGGCAAGCACCGTGCCATCTCTATTTCGGAGGAGAAAGGAGACATTTGGTGCGAAAAGACTGATGTAGCAATCAGTGGACCGGAGTTCTACCGCACTTGCTATGGGTTGTTGCAGGACCCGAAGCCTAACTCGGAGGCAGATTCTTGTTGTACTCGTTGGGTAGACGGCGTTGTGGTACAATCTGACGGGTGCTTCAAATCTTGGAGACCGT |
| Avr1 | ATCTTCTCACTCTACGCTTCCAAAGGGGGAGGAGGGTGACATCATCGGAACATTCAACTTTAGCAGCTCAGACAGTCAGCCGCTTAAAATCCACTGGGTAGATACTCCGGATAGTTCAGGCTCCAATCTCGTTAAAAGATCTGCTCATACCGAGTCTGTTTGTGTACATGCTGGCACTGCTACTGGCGCAGATCTCCACTGGCTTAATGCAATATGCACTGGTAAATCGACATATACTGTTAACTGCGCGCCTGCGGGTAATAAAAATGCTGGATCGACACACACCGGAACATGTCCCGCAGGTCAGGATTGCTTCCAGTTGGAGCAAGTTGGCAACTTTTGGGGTGATAGAGAGCCTGACGCTACTTGCTCTCCGTCTAACACTGTTTTTGATGCGGTAGATGACAAGGAGGCAACACATGTAAATGGCAAAGTTGTCACGCGTGCAGGAAAGCCAGGAATAGGACGAAAGCTCATTCGACTCAAGGCCCAAGTCTATCGCAGAGACGGGCATTATGGACAAACTTCAAGAATGGGCTTTTTCCGTAATGGTAAGGAGGTATACCACATTGATAATGTGGCAAGCATGGAACCGACATGGAACTTTGATCCTTCCAGCGACCAGAGCTTTAGCTTCTTCTTCACTCCGGGGCCTAATGCATTTCGCATTCAAGGTACACTTAACTTAGCTTAGGTAATTTGGAGAGGA |
| Avr1^ADVKT^ | ATCTTCTCACTCTACGCTTCCAAAGGGGGAGGAGGGTGACATCATCGGAACATTCAACTTTAGCAGCTCAGACAGTCAGCCGCTTAAAATCCACTGGGTAGATACTCCGGATAGTTCAGGCTCCAATCTCGTTAAAAGATCTGCTCATACCGAGTCTGTTTGTGTACATGCTGGCACTGCTACTGGCGCAGATCTCCACTGGCTTAATGCAATATGCACTGGTAAATCGACATATACTGTTAACTGCGCGCCTGCGGGTAATAAAAATGCTGGATCGACACACACCGGAACATGTCCCGCAGGTCAGGATTGCTTCCAGTTGGAGCAAGTTGGCAACTTTTGGGGTGATAGAGAGCCTGACGCTACTTGCTCTCCGTCTAACACTGTTTTTGATGCGGCCGACGTGAAGACCGCAACACATGTAAATGGCAAAGTTGTCACGCGTGCAGGAAAGCCAGGAATAGGACGAAAGCTCATTCGACTCAAGGCCCAAGTCTATCGCAGAGACGGGCATTATGGACAAACTTCAAGAATGGGCTTTTTCCGTAATGGTAAGGAGGTATACCACATTGATAATGTGGCAAGCATGGAACCGACATGGAACTTTGATCCTTCCAGCGACCAGAGCTTTAGCTTCTTCTTCACTCCGGGGCCTAATGCATTTCGCATTCAAGGTACACTTAACTTAGCTTAGGTAATTTGGAGAGGA |
| Avr1^EEEYGIN^ | ATCTTCTCACTCTACGCTTCCAAAGGGGGAGGAGGGTGACATCATCGGAACATTCAACTTTAGCAGCTCAGACAGTCAGCCGCTTAAAATCCACTGGGTAGATACTCCGGATAGTTCAGGCTCCAATCTCGTTAAAAGATCTGCTCATACCGAGTCTGTTTGTGTACATGCTGGCACTGCTACTGGCGCAGATCTCCACTGGCTTAATGCAATATGCACTGGTAAATCGACATATACTGTTAACTGCGCGCCTGCGGGTAATAAAAATGCTGGATCGACACACACCGGAACATGTCCCGCAGGTCAGGATTGCTTCCAGTTGGAGCAAGTTGGCAACTTTTGGGGTGATAGAGAGCCTGACGCTACTTGCTCTCCGTCTAACACTGTTTTTGATGCGGTAGATGACAAGGAGGCAACACATGTAAATGGCAAAGTTGTCACGCGTGCAGGAAAGCCAGGAATAGGACGAAAGCTCATTCGACTCAAGGCCCAAGTCTATCGCAGAGACGGGCATTATGGACAAACTTCAAGAATGGGCTTTTTCCGTAATGGTGAGGAAGAGTATGGTATTAACAATGTGGCAAGCATGGAACCGACATGGAACTTTGATCCTTCCAGCGACCAGAGCTTTAGCTTCTTCTTCACTCCGGGGCCTAATGCATTTCGCATTCAAGGTACACTTAACTTAGCTTAGGTAATTTGGAGAGGA |
| Avr1^IDH^ | ATCTTCTCACTCTACGCTTCCAAAGGGGGAGGAGGGTGACATCATCGGAACATTCAACTTTAGCAGCTCAGACAGTCAGCCGCTTAAAATCCACTGGGTAGATACTCCGGATAGTTCAGGCTCCAATCTCGTTAAAAGATCTGCTCATACCGAGTCTGTTTGTGTACATGCTGGCACTGCTACTGGCGCAGATCTCCACTGGCTTAATGCAATATGCACTGGTAAATCGACATATACTGTTAACTGCGCGCCTGCGGGTAATAAAAATGCTGGATCGACACACACCGGAACATGTCCCGCAGGTCAGGATTGCTTCCAGTTGGAGCAAGTTGGCAACTTTTGGGGTGATAGAGAGCCTGACGCTACTTGCTCTCCGTCTAACACTGTTTTTGATGCGGTAGATGACAAGGAGGCAACACATGTAAATGGCAAAGTTGTCACGCGTGCAGGAAAGCCAGGAATAGGACGAAAGCTCATTCGACTCAAGGCCCAAGTCTATCGCAGAGACGGGCATTATGGACAAACTTCAAGAATGGGCTTTTTCCGTAATGGTAAGGAGGTATACCACATTGATAATGTGGCAAGCATGGAACCGACATGGAACTTTGATCCTTCGATTGATCACTCGTTTAGCTTCTTCTTCACTCCGGGGCCTAATGCATTTCGCATTCAAGGTACACTTAACTTAGCTTAGGTAATTTGGAGAGGA |
| Avr1^NGQAR^ | ATCTTCTCACTCTACGCTTCCAAAGGGGGAGGAGGGTGACATCATCGGAACATTCAACTTTAGCAGCTCAGACAGTCAGCCGCTTAAAATCCACTGGGTAGATACTCCGGATAGTTCAGGCTCCAATCTCGTTAAAAGATCTGCTCATACCGAGTCTGTTTGTGTACATGCTGGCACTGCTACTGGCGCAGATCTCCACTGGCTTAATGCAATATGCACTGGTAAATCGACATATACTGTTAACTGCGCGCCTGCGGGTAATAAAAATGCTGGATCGACACACACCGGAACATGTCCCGCAGGTCAGGATTGCTTCCAGTTGGAGCAAGTTGGCAACTTTTGGGGTGATAGAGAGCCTGACGCTACTTGCTCTCCGTCTAACACTGTTTTTGATGCGGTAGATGACAAGGAGGCAACACATGTAAATGGCAAAGTTGTCACGCGTGCAGGAAAGCCAAACGGTCAGGCTCGCCTCATTCGACTCAAGGCCCAAGTCTATCGCAGAGACGGGCATTATGGACAAACTTCAAGAATGGGCTTTTTCCGTAATGGTAAGGAGGTATACCACATTGATAATGTGGCAAGCATGGAACCGACATGGAACTTTGATCCTTCCAGCGACCAGAGCTTTAGCTTCTTCTTCACTCCGGGGCCTAATGCATTTCGCATTCAAGGTACACTTAACTTAGCTTAGGTAATTTGGAGAGGA |
| FonSIX4 | ATCTTCTCACTCTACGCTGCCGACCGATGAGGGGGATATTATCGGCACTTTTTCAATCGACAGTTCTGACTCACAGCCTATCAAGGTCCGCTGGTTAGATACGCCAGAGATTTCTGGACGTTCGTTAGTCAAACGCTCGGCCTTGAGCCAGTCTGTCTGCACTCATGCTGGAACGGCGACGGGTGCCGATCTTCATTGGCTGAATGGGATTTGCACTGGGAAATCAACCTATACTGTGAATTGTGCTCCCGCAGGTAACAAAAATGCGGGGAGCTCCCACACCGGCACATGCCCACCGGACGAAGACTGTTTCCAACTGGAGCGCGTGGGCAATTTCTGGGGCGACTTGGAACCCGATGTAACCTGTGCAAAGTCGGGCACAGTCTTTGACGCGGCCGACGTGAAGACCGCGACCCACGTTAACGGCAAAATTGTGACCCGTGCAGGAAAGCCTAACGGTCAGGCTCGCCTGATCCGCCTTAAAGCCCAAGTCTACCGCCGCGATGGTCATTATGGTCAGACCAGTCGCATGGGTTTCTTTCGTAACGGGGAGGAAGAGTATGGTATTAACAACGTGGCATCCATGGGGCCGACTTGGAATTTCGATCCTTCGATTGATCACTCGTTCAGTTTCTTCTTCACGCCTGGGCCAGATGCATTCCGCGTACAGGGGACCATTAATTTAGCGTAGGTAATTTGGAGAGGA |
| FonSIX4^KEVYHID^ | ATCTTCTCACTCTACGCTGCCGACCGATGAGGGGGATATTATCGGCACTTTTTCAATCGACAGTTCTGACTCACAGCCTATCAAGGTCCGCTGGTTAGATACGCCAGAGATTTCTGGACGTTCGTTAGTCAAACGCTCGGCCTTGAGCCAGTCTGTCTGCACTCATGCTGGAACGGCGACGGGTGCCGATCTTCATTGGCTGAATGGGATTTGCACTGGGAAATCAACCTATACTGTGAATTGTGCTCCCGCAGGTAACAAAAATGCGGGGAGCTCCCACACCGGCACATGCCCACCGGACGAAGACTGTTTCCAACTGGAGCGCGTGGGCAATTTCTGGGGCGACTTGGAACCCGATGTAACCTGTGCAAAGTCGGGCACAGTCTTTGACGCGGCCGACGTGAAGACCGCGACCCACGTTAACGGCAAAATTGTGACCCGTGCAGGAAAGCCTAACGGTCAGGCTCGCCTGATCCGCCTTAAAGCCCAAGTCTACCGCCGCGATGGTCATTATGGTCAGACCAGTCGCATGGGTTTCTTTCGTAACGGGAAGGAGGTATACCACATTGATAACGTGGCATCCATGGGGCCGACTTGGAATTTCGATCCTTCGATTGATCACTCGTTCAGTTTCTTCTTCACGCCTGGGCCAGATGCATTCCGCGTACAGGGGACCATTAATTTAGCGTAGGTAATTTGGAGAGGA |
| *Gene sequences have been codon optimised for expression in *E. coli*. Cloning overhangs have been underlined. | |
