# Supplementary material for: The structural repertoire of Fusarium oxysporum f. sp. lycopersici effectors revealed by experimental and computational studies
Source: eLife. 2024 Feb 27;12:RP89280. doi: 10.7554/eLife.89280 (PMC10942635; doi:10.7554/eLife.89280)
Supplement: Supplementary file 5. [file elife-89280-supp5.docx]

**S5 Table.** Primers used in this study

| **Name** | **Sequence (5’- 3’)** |
| --- | --- |
| Avr1_Fw | TAGGTCTCCAATGCTTCCAAAGGGGGAGGAGGGTG |
| Avr1_Rv | ACGGTCTCCAAGAAGCTAAGTTAAGTGTACCTTGAATGCGA |
| Avr3_Fw | TAGGTCTCCAATGCAAGAGGCTGCGGTTCGGGA |
| Avr3_Rv | ACGGTCTCCAAGAGGCGTTTGGATATACCAGCCCACAC |
| IF-Avr1_Fw | ATCTTCTCACTCTACGCTTCCAAAGGGGGAGG |
| IF-Avr1_Rv | TCCTCTCCAAATTACCTAAGCTAAGTTAAGTGTACCTTGA |
| IF-Avr1-Ctag_Rv | GCTTCCGCTAGCTAAGTTAA |
| IF-Avr1_3xHA_Fw | TTAGCTAGCGGAAGCTACCCATATGACGTTCCA |
| IF-Avr1-N_Rv | TCCTCTCCAAATTACCTACTAGTTAGACGGAGAGCAAGTAG |
| IF-Avr1-C_Fw | ATCTTCTCACTCTACGACTGTTTTTGATGCGGTAGAT |
| IF-Avr1-N-Ctag_Rv | GCTGTTAGACGGAGAGC |
| IF-Avr1-N_3xHA_Fw | TCTCCGTCTAACAGCTACCCATATGACGTTCCA |
| IF-FonSIX4_Fw | ATCTTCTCACTCTACGCTGCCGACCGATGAG |
| IF-FonSIX4_Rv | TCCTCTCCAAATTACCTACGCTAAATTAATGGTCCCC |
| IF-FonSIX4-Ctag_Rv | GCTTCCGCTCGCTAAATTA |
| IF-FonSIX4_3xHA_Fw | TTAGCGAGCGGAAGCTACCCATATGACGTTCCA |
| IF-FonSIX4-N_Rv | TCCTCTCCAAATTACCTACTAGCCCGACTTTGCACAGG |
| IF-FonSIX4-C_Fw | ATCTTCTCACTCTACGACAGTCTTTGACGCGG |
| IF-FonSIX4-N-Ctag_Rv | GCTGCCCGACTTTGC |
| IF-FonSIX4-N_3xHA_Fw | GCAAAGTCGGGCAGCTACCCATATGACGTTCCA |
| IF-3xHA_RvNew | TCCTCTCCAAATTACCTAGTAGTCAGGTACGTCGTAAG |
